# Targeting KIF20A blocks lactylation modification to suppress immune escape in hepatocellular carcinoma

**DOI:** 10.1016/j.isci.2026.115372

**Published:** 2026-03-13

**Authors:** Shujia Chen, Lili Zhao, Tongguo Miao, Ping Han, Jie Liu, Jiancun Hou, Qiang Zhao, Fengmei Wang, Jia Li

**Affiliations:** 1 Department of Hepatology and Gastroenterology, Tianjin First Central Hospital, 2 West Baoshan Road, Xiqing District, Tianjin 300392, China; 2 Department of Hepatology and Gastroenterology, Tianjin Second People’s Hospital, No. 7, Sudi South Road, Nankai District, Tianjin 300192, China; 3 Department of Surgery, Tianjin Second People’s Hospital, Tianjin, China; 4 State Key Laboratory of Medicinal Chemical Biology, Key Laboratory of Bioactive Materials (Ministry of Education), Frontiers Science Center for Cell Responses, College of Life Sciences, Nankai University, Tianjin 300071, China

**Keywords:** health sciences, medicine, medical specialty, internal medicine, hepatology

## Abstract

Hepatocellular carcinoma (HCC) evades anti-PD-1 immunotherapy via an immunosuppressive microenvironment, where lactate links metabolic reprogramming to epigenetic regulation. We analyzed pan-lysine lactylation and H3K18 lactylation (H3K18la) in 89 HCC patient pairs, and validated functional mechanisms using glycolysis inhibition, HCC-CD8^+^ T cell co-cultures, and rescue assays. *In vivo* efficacy was assessed in subcutaneous and orthotopic HCC mouse models. H3K18la levels were elevated in HCC, correlating with advanced staging and poor prognosis. Lactate induced H3K18la to transcriptionally upregulate KIF20A, which stabilized the c-Myc/PD-L1 axis and suppressed cytotoxic T cell function. Combined glycolysis inhibition and anti-PD-1 therapy reversed this immunosuppression and synergistically inhibited tumor growth. This study identifies an H3K18la-KIF20A/PD-L1 axis as a key metabolic-epigenetic checkpoint, highlighting glycolysis targeting as a promising strategy to enhance anti-PD-1 responses in HCC.

## Highlights

• H3K18la serves as a key epigenetic driver for HCC immune evasion by targeting KIF20A
• Depletion of KIF20A reverses lactic acid-induced progression and immunosuppression of HCC
• H3K18la-KIF20A/c-Myc/PD-L1 axis links metabolism, epigenetics, and antitumor immunity in HCC

## Graphical abstract

**Figure:**
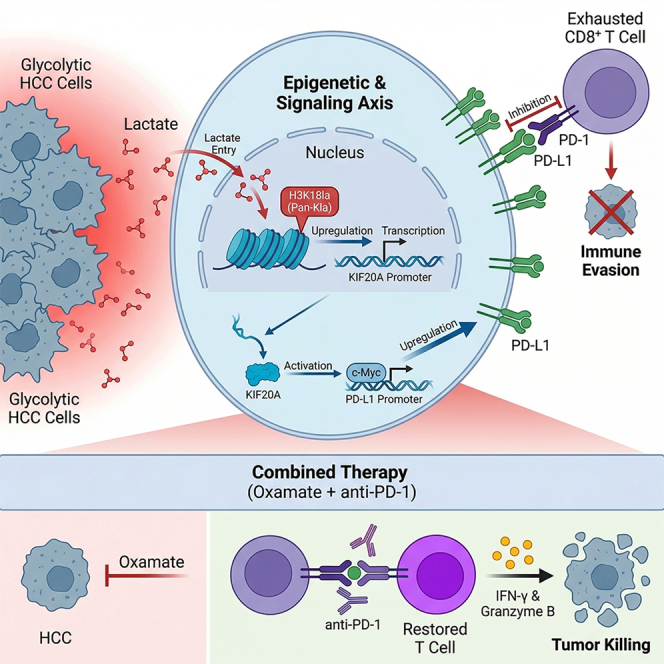

## Introduction

Hepatocellular carcinoma (HCC) poses a growing global health burden, characterized by rising incidence, mortality, and limited late-stage therapeutic options.^1^ While immune checkpoint inhibitors, notably anti-PD-1 agents, have transformed cancer care, their efficacy in HCC is frequently compromised by tumor microenvironment (TME)-driven immune evasion. Emerging evidence suggests metabolic reprogramming and epigenetic deregulation synergize to drive immunosuppression, yet their molecular crosstalk in HCC remains poorly understood.^2^^,^^3^ Studies have shown that glycolysis-induced lactate accumulation correlates with T cell exhaustion,^4^^,^^5^ but mechanistic insights into how metabolic byproducts reprogram epigenetic landscapes to enforce immune tolerance are scarce. This gap underscores the urgency of dissecting tissue-specific pathways integrating metabolism, epigenetics, and immunity in HCC.

As a key glycolytic metabolite, lactate not only modulates intracellular signaling, oxidative stress responses, lactate shuttling, and lactylation modifications in tumor cells, but also profoundly shapes the immune microenvironment.^6^ Lactate-mediated crosstalk with immune cells regulates immune responses, metabolic adaptation, and drug resistance.^7^^,^^8^ Lysine lactylation (Kla) of histones has been successively reported in cancers,^9^^,^^10^^,^^11^^,^^12^ with particular emphasis on histone H3 lysine 18 lactylation (H3K18la). Studies have shown that H3K18la is involved in multiple biological processes such as tumor initiation and progression,^9^^,^^13^^,^^14^ immune evasion,^15^^,^^16^ and metabolic reprogramming of cancer cells.^17^^,^^18^ Yang et al. reported that VHL inactivation triggers a cascade in clear cell renal cell carcinoma, where H3K18la-mediated upregulation of PDGFRβ creates a positive feedback loop, thereby accelerating cancer progression.^18^ Li et al. illustrated that in CRC, H3K18la hyper-lactylation transcriptionally activates Rubicon-like protein, augmenting autophagosome maturation and fueling tumor progression.^19^ Despite these advances in other malignancies, the target genes of H3K18la and its precise role in immune evasion within the HCC microenvironment remain largely unexplored.

Previous ChIP-seq analysis identified kinesin family member 20A (KIF20A) as a key gene bound by H3K18la,^20^ although the functional relationship between them has not been fully elucidated. KIF20A, a key player in cell division and intracellular transport as a mitotic kinesin, has demonstrated significant prognostic value across multiple cancer types.^21^^,^^22^^,^^23^^,^^24^^,^^25^ Our earlier work demonstrated that targeting KIF20A induces mismatch repair deficiency by enhancing c-Myc ubiquitination, thereby exposing more antigens in tumor cells and improving the efficacy of PD-1 inhibitors against HCC.^26^ While c-Myc’s role in promoting PD-L1 expression via direct promoter binding is a recognized immune evasion mechanism, the potential functional linkage between KIF20A and this axis, along with H3K18la′s regulatory role through KIF20A, constitutes a significant knowledge gap requiring investigation.

This study elucidates how the H3K18la-KIF20A axis drives immune evasion in HCC through metabolic-epigenetic crosstalk. We found that H3K18la levels predict responses to anti-PD-1 therapy and mechanistically showed that H3K18la transcriptionally activates KIF20A to promote c-Myc-dependent PD-L1 expression. Furthermore, glycolytic inhibition synergized with anti-PD-1 treatment, providing a rationale for targeting this axis to overcome immunotherapy resistance.

## Results

### Upregulated Pan Kla and H3K18la expressions are linked to adverse outcomes among patients with HCC

**Figure 1:**
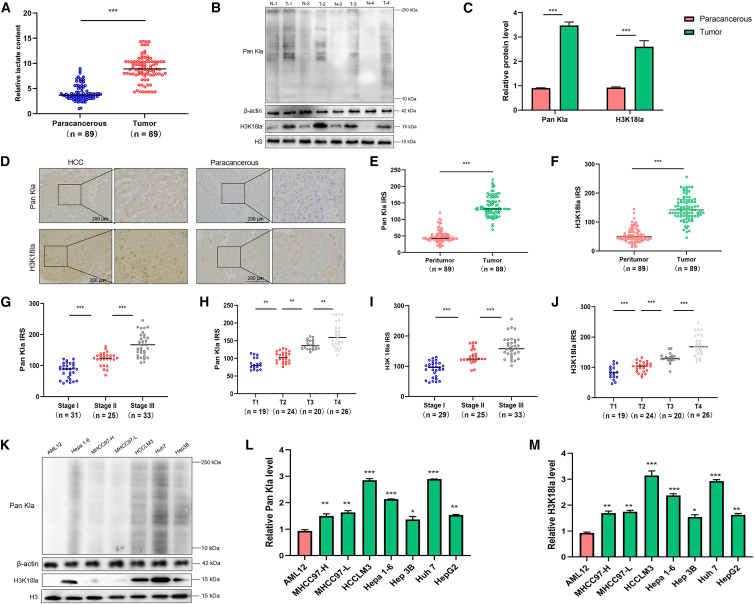
Pan Kla is upregulated in HCC and associated with disease progression (A) The scatterplot shows the overall lactate content in 89 pairs of HCC (*n* = 89) and paracanerous tissues (*n* = 89). (B) Representative WB images of Pan Kla, H3K18la, and H3 (loading control) in paired samples. (C) Quantitative analysis of WB data for Pan Kla and H3K18la. (D) IHC staining of Pan Kla and H3K18la in HCC and paracancerous tissues (scale bars, 100 μm). (E and F) Statistical analysis of IHC results, presented as average optical density (E) and positive rate (F). (G–J) Expression of Pan Kla and H3K18la stratified by TNM stages (stage I: *n* = 29; stage II: *n* = 25; stage III: *n* = 33). Data are shown as scatterplots of relative levels. (K) WB analysis of Pan Kla, H3K18la, and H3 in normal liver cell line (AML12) and various HCC cell lines. (L and M) Quantitative comparison of Pan Kla (L) and H3K18la (M) levels in HCC cell lines versus AML12. All data are presented as mean ± SD. Statistical significance: ∗∗∗*p* < 0.001, ∗∗*p* < 0.01, and ∗*p* < 0.05 (unpaired *t* test for paired tissues; one-way ANOVA for stage and cell line comparisons). Experiments were repeated three times.

To investigate the potential value of lactylation modification in HCC, we measured lactate concentrations in HCC tissues and paracancerous tissues, and found that lactate levels were significantly higher in HCC tissues than in paired paracancerous tissues (Figure 1A). To evaluate the role of H3K18la in HCC progression, we first analyzed Pan Kla and H3K18la in 89 pairs of HCC and adjacent paracancerous tissues. Quantitative immunoblotting (Figures 1B and 1C) revealed a marked increase in Pan Kla and H3K18la levels within HCC tumors compared to matched paracancerous samples. Consistent with these findings, IHC staining (Figures 1D–1F) showed stronger positive staining for Pan Kla and H3K18la in HCC tissues, with higher average optical density and positive rates relative to paracancerous tissues. These data suggest a potential oncogenic relevance of Pan Kla and H3K18la in HCC.

We further stratified patients by TNM stages and analyzed lactylation levels. As shown in Figures 1G–1J, the levels of Pan Kla and H3K18la gradually increased with the progression of HCC stages. Specifically, stage III tumors exhibited the highest levels of Pan Kla and H3K18la, whereas stage I tumors showed the lowest. This trend indicates that Pan Kla and H3K18la may be involved in HCC progression and could serve as potential indicators of disease progression. To validate the cell-specific pattern, we measured lactylation levels in a panel of HCC cell lines, using the normal liver cell line AML12 as a control. WB analysis (Figures 1K–1M) demonstrated that the levels of Pan Kla and H3K18la were higher in HCC cells compared to normal AML12 cells.

Collectively, our results indicate a robust correlation between elevated Pan Kla/H3K18la levels and adverse prognosis among patients with HCC.

### Blocking H3K18 lactylation via glycolysis inhibition in HCC cells restores the function of co-cultured CD8^+^ T cells

**Figure 2:**
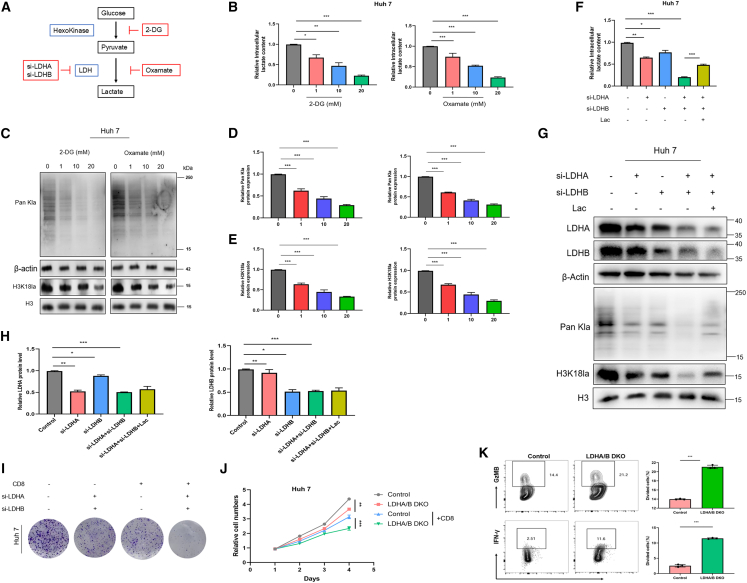
Glycolysis inhibition-mediated blockade of H3K18la in HCC cells restores the function of co-cultured CD8^+^ T cells (A) Schematic diagram of the experimental design. (B) Changes in lactate levels in Huh7 cells after treatment with 2-DG or oxamate. (C–E) Representative WB results showing the levels of Pan Kla and H3K18la in Huh7 cells treated with inhibitors. (F) Lactate levels in DKO cells with or without lactate supplementation. (G and H) WB analysis of the protein levels of LDHA, LDHB, Pan Kla, and H3K18la in DKO cells with or without lactate treatment. H3 and β-actin were used as loading controls for lactylation markers and LDH isoforms, respectively. (I) Colony formation assays show the proliferation of HCC cells (control group vs. DKO group) cultured alone or co-cultured with CD8^+^ T cells. (J) Quantitative analysis of the proliferation of co-cultured CD8^+^ T cells by FC. (K) FC analysis of the expression levels of GzMB (L) and IFN-γ (M) in co-cultured CD8^+^ T cells. All data are shown as mean ± SD. (B, D, E, and G) were analyzed using one-way ANOVA, (J) analyzed by two-way ANOVA, and (K) was analyzed by Student’s *t* test. ∗*p* < 0.05, ∗∗*p* < 0.01, and ∗∗∗*p* < 0.001. Experiments were repeated three times.

Tumor-derived lactate significantly suppresses immune cell function. We further explored whether changes in histone lactylation within HCC could induce dysfunction in cytotoxic T lymphocytes (CTLs). Initially, treating Huh7 cells with the glycolytic inhibitors 2-DG and oxamate led to a dose-dependent decrease in intracellular lactate levels (Figures 2A and 2B), paralleled by a simultaneous reduction in Pan Kla and H3K18la levels (Figures 2C–2E).

Lactate dehydrogenase (LDH), an essential glycolytic enzyme, consists of two major subunit types: LDHA and LDHB. Notably, compared to single knockdowns of LDHA or LDHB and other control groups, LDHA/B DKO substantially decreased intracellular lactate levels (Figure 2F) and significantly lowered the protein expression of both Pan Kla and H3K18la (Figures 2G and 2H). Subsequently, we co-cultured Huh.

7 cells (control or LDHA/B DKO group) with CD8^+^ T cells *in vitro*. Colony formation and CCK-8 assays demonstrated that LDHA/LDHB double knockdown alone inhibited HCC cell proliferation; this inhibition on colony formation and proliferation became more prominent when co-cultured with CD8^+^ T cells (Figures 2I and 2J). Additionally, compared with controls, LDHA/LDHB DKO in HCC cells significantly restored the proliferation of co-cultured CD8^+^ T cells and upregulated GzMB and IFN-γ expression in CD8^+^ T cells (Figure 2K).

Our data reveal that inhibiting H3K18la restores CD8^+^ T cell effector activity *in vitro*.

### Blocking lactate-derived H3K18 lactylation suppresses HCC immune evasion by enhancing CTL cytotoxicity

**Figure 3:**
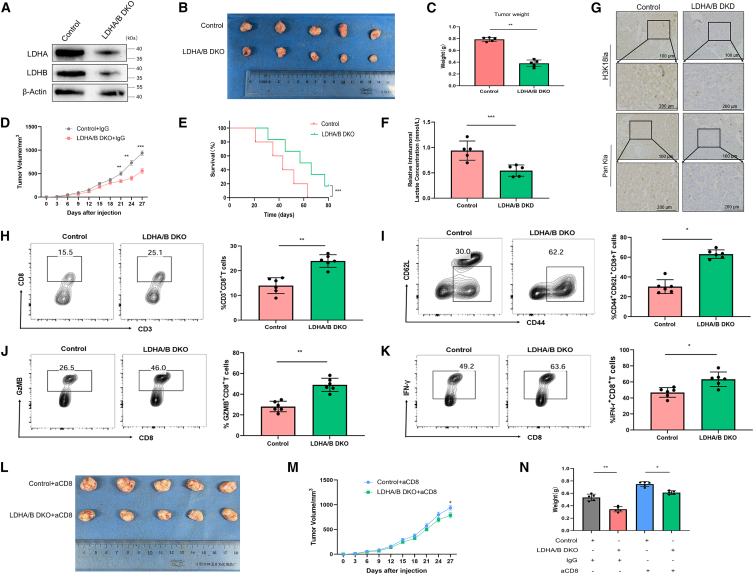
Knockdown of LDHA/B inhibits HCC growth by activating CD8^+^ T cell-mediated antitumor immunity (A) WB analysis of LDHA, LDHB, and β-actin (loading control) expression in control and LDHA/B DKO Hepa 1–6 cells. (B) Representative images of tumors dissected from tumor-bearing mice (*n* = 6). Tumor weights (C), tumor growth curves (D), Kaplan-Meier survival curve (*n* = 6) (E), and lactate contents in tumor tissues (F) of the control and LDHA/B DKO groups (*n* = 6). (G) IHC staining of H3K18la and Pan Kla in tumor tissues (scale bars, 100 μm, *n* = 6). (H–M) FC analysis of tumor-infiltrating immune cells: TILs were isolated from control and LDHA/B-DKO tumors. FC plots (left) and quantitative statistics (right) show: the proportion of CD3^+^CD8^+^ CTLs (H), the proportion of CD44hiCD62Llo effector memory CTLs (I), and the proportions of intratumoral GzMB^+^ (J), and IFN-γ^+^ (K) CTLs with representative scatterplots (*n* = 6). Cell aggregates and debris were first excluded, followed by gating on the target cell populations and markers of interest. Representative FC plots and quantitative data are presented. (L and M) Tumor size and growth curves of mice treated with anti-CD8 antibody to deplete CD8^+^ T cells or isotype control (IgG) (*n* = 6). (N) Mouse body weights at the end of the experiment (*n* = 6). Data are presented as mean ± SD. (C–F, and H–M) were analyzed using Student’s *t* test, and (N) was analyzed by two-way ANOVA. ∗*p* < 0.05, ∗∗*p* < 0.01, and ∗∗∗*p* < 0.001; ns, not significant. All experiments were repeated three times. β-actin served as the loading control for WB experiments.

Previous studies have shown that excessive lactate within tumors can foster an immunosuppressive microenvironment in HCC.^27^ To explore whether lactate-derived H3K18la contributes to CTL dysfunction in HCC *in vivo*, we employed shRNA to establish LDHA/B DKO in HCC cells (Figure 3A). After injecting control or LDHA/B DKO HCC cells subcutaneously into mice, we observed that mice receiving LDHA/B DKO cells developed significantly smaller tumors and lived longer than the control group, as clearly shown in Figures 3B–3E.

Further analysis of the remodeling effect of glycolysis blockade on the TME revealed that LDHA/B DKO significantly decreased lactate concentration in tumor tissues (Figure 3F). IHC assessment revealed a parallel decline in the levels of Pan Kla and H3K18la (Figure 3G), providing further evidence that lactate serves as a primary inducer of H3K18 lactylation in HCC cells, thereby facilitating tumor advancement. Following this, FC analysis demonstrated that both the proportion and functionality of CTLs were markedly elevated in LDHA/B-DKO tumors relative to controls: The percentage of CD3^+^CD8^+^ CTLs was significantly increased (Figure 3H), as was the proportion of effector memory CTLs (CD44hiCD62Llo; Figure 3I). Additionally, the percentages of CTLs expressing GzMB^+^ (Figure 3J) and IFN-γ^+^ (Figure 3L) were all significantly elevated. To substantiate the critical role of cytotoxic T cells, *in vivo* CD8^+^ T cell depletion reversed the tumor-suppressive effects of LDHA/B knockout (Figures 3L–3N), demonstrating that the antitumor mechanism is immune-mediated.

In conclusion, our findings demonstrate that blocking lactate-derived H3K18la reshapes the tumor immune microenvironment and enhances CTL cytotoxicity, thereby suppressing HCC immune evasion.

### H3K18la activates the transcription of KIF20A in HCC cells

Histone lactylation, a newly identified post-translational modification, reportedly directly drives gene transcription. Emerging evidence indicates that KIF20A serves as a target gene for H3K18la.^20^ Notably, our previous work has confirmed that KIF20A is significantly associated with poor prognosis in HCC, and that targeting KIF20A enhances the efficacy of anti-PD-1 therapy by promoting c-Myc ubiquitination and inducing mismatch repair deficiency.^26^ Metabolic reprogramming and epigenetic dysregulation are known to synergistically drive immunosuppression in HCC, leading to suboptimal responses to PD-1 inhibitors in patients; however, the molecular interplay between these pathways in HCC remains poorly understood.

**Figure 4:**
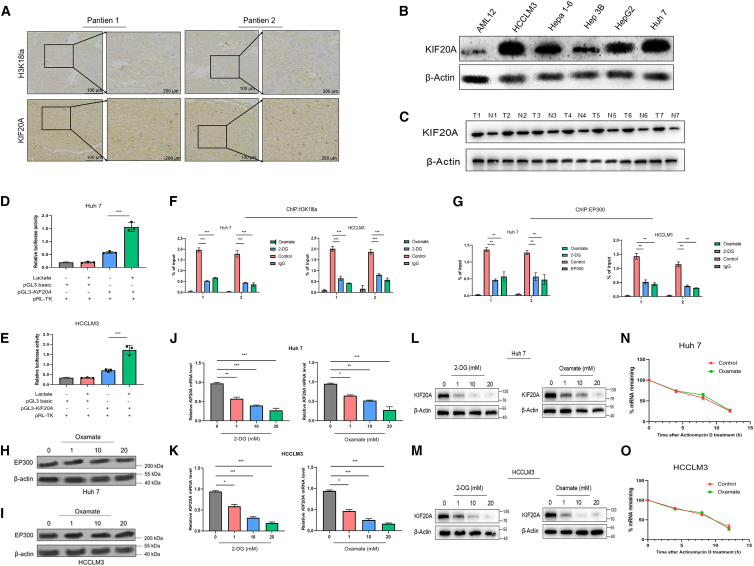
H3K18la activates transcription of KIF20A in HCC cells (A) Representative IHC images of KIF20A and H3K18la expression in the HCC cohort and correlation analysis. (B) WB analysis of KIF20A protein levels in normal liver cell line (AML12) and HCC cell lines. (C) Detection and quantitative analysis of KIF20A protein expression in clinical HCC tissues and adjacent non-tumor tissues. (D and E) pRL-TK was co-transfected with PGL3-KIF20A or empty PGL3-basic plasmid into Huh7 and HCCLM3 cells, and luciferase activity was measured after treatment with 20 mM lactate or no treatment for 24 h. (F) After HCC cells were treated with 2-DG or oxamate for 24 h, the binding of H3K18la to the KIF20A promoter region was detected by ChIP-PCR. (G) Huh7 and HCCLM3 cells were incubated with 2-DG or oxamate for 24 h. The binding status of EP300 to the KIF20A promoter region was determined by ChIP-PCR. (H and I) EP300 protein expression in Huh7 and HCCLM3 cells was measured by WB after treatment with oxamate for 24 h mRNA expression levels of KIF20A in Huh7 (J) and HCCLM3 cells (K) after treatment with 2-DG or oxamate for 24 h. Protein expression levels of KIF20A in Huh7 cells (L) and HCCLM3 cells (M) after treatment with 2-DG or oxamate for 24 h. (N) Huh7 and HCCLM3 (O) cells were treated with 20 mM oxamate or solvent control for 24 h, then stimulated with actinomycin D for the indicated times, and KIF20A mRNA expression levels were detected by RT-PCR. Data are presented as mean ± SD and analyzed by one-way ANOVA (F, G, J, and K), and two-way ANOVA (D and E). ∗∗∗*p* < 0.001. All experiments were repeated three times.

Upregulation of KIF20A protein was confirmed by immunoblotting in both HCC-derived cell lines and patient tissues (Figures 4A–4C), with a positive correlation with H3K18la levels. Among them, Huh7 and HCCLM3 exhibited the highest expression among multiple HCC cell lines. Therefore, subsequent studies were conducted based on these two cell lines. To delve into the regulatory mechanism between KIF20A and H3K18la expression in HCC, we generated a luciferase reporter vector harboring the KIF20A promoter. The results demonstrated that lactate substantially boosted luciferase activity driven by this promoter (Figures 4D and 4E). Further confirmation through ChIP-PCR indicated that 2-DG and oxamate notably hindered the binding of H3K18la and the lactyltransferase EP300 to the KIF20A promoter (Figures 4F and 4G), while EP300 expression stayed constant following glycolysis inhibition (Figures 4H and 4I). Additionally, 2-DG and oxamate decreased KIF20A mRNA and protein levels in a dose-dependent fashion (Figures 4J–4M); however, after actinomycin D treatment, KIF20A mRNA levels in the oxamate group were similar to those in the control group (Figures 4N and 4O), suggesting that glycolysis inhibition reduces KIF20A expression via transcriptional regulation rather than altering mRNA stability.

In summary, H3K18la positively modulates KIF20A transcriptional expression by directly interacting with its promoter region.

### KIF20A promotes the immune evasion of HCC cells by regulating PD-L1 expression via c-myc

**Figure 5:**
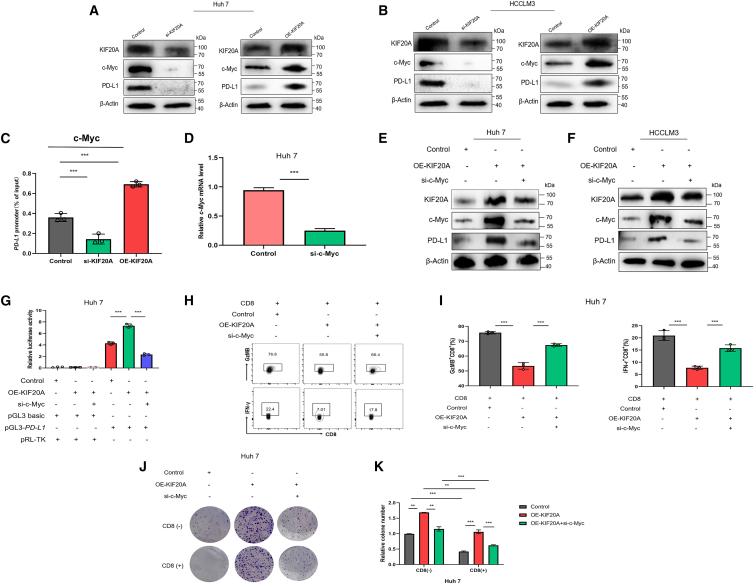
KIF20A regulates PD-L1 expression via c-Myc (A and B) Huh7 and HCCLM3 cells were transfected with KIF20A small interfering RNAs (siRNAs) or KIF20A overexpression (OE) pcDNA3.1 plasmids, respectively. The protein levels of KIF20A, c-Myc, and PD-L1 were measured by WB in Huh7 and HCCLM3 cells. (C) ChIP-PCR was performed to assess the binding of c-Myc to the PD-L1 promoter region in Huh7 cells. (D) Huh7 cells were transfected with c-Myc siRNAs, and the knockdown efficiency of c-Myc was verified by RT-PCR. (E and F) Huh7 and HCCLM3 cells were co-transfected with KIF20A OE plasmids and c-Myc siRNAs. The protein levels of KIF20A, PD-L1, and c-Myc were determined by WB. (G) Huh7 cells were co-transfected with pGL3-PD-L1 or pGL3-basic plasmids, KIF20A OE pcDNA3.1 plasmids, c-Myc siRNAs, and pRL-TK plasmids, followed by luciferase activity assay. (H and I) KIF20A promotes HCC cell immune evasion by regulating PD-L1 expression via c-Myc. HCC cells from different groups were co-cultured with CD8^+^ T cells. Representative FC plots and quantitative analysis of GzMB and IFN-γ expression in CD8^+^ T cells co-cultured with Huh7 cells. (J and K) Representative images and quantitative analysis of colony formation in Huh7 cells with or without CD8^+^ T cell co-culture. Data are presented as mean ± SD. Statistical analyses were performed using Student’s *t* test (D), one-way ANOVA (C, I), or two-way ANOVA (G, K). ∗∗*p* < 0.01 and ∗∗∗*p* < 0.001. All experiments were repeated three times.

To avoid detection by the body’s immune system, cancer cells produce various molecules that suppress immune responses, with PD-L1 standing out as a particularly important one because of its key role in controlling immune activity. First, we knocked down or overexpressed KIF20A in the Huh7 cell line, respectively. Both RT-PCR and WB assays showed that KIF20A knockdown significantly decreased the expression of c-Myc and PD-L1, whereas KIF20A overexpression increased their levels (Figures 5A–5C). Given that the oncogenic effect of KIF20A is crucial for the malignant progression of HCC and that c-Myc is a known regulator of PD-L1, we further explored whether KIF20A regulates PD-L1 expression through c-Myc. We observed the bidirectional modulation of c-Myc/PD-L1 promoter binding by KIF20A-knockdown diminished, whereas overexpression potentiated this interaction (Figure 5D). We further co-transfected KIF20A-overexpressing plasmids with c-Myc siRNAs into Huh7, and found that c-Myc knockdown partially reversed the KIF20A overexpression-induced upregulation of PD-L1 (Figures 5E and 5F). Luciferase reporter assays further confirmed that KIF20A overexpression enhanced PD-L1 promoter-driven luciferase activity, while c-Myc knockdown significantly inhibited this activity (Figure 5G).

In co-culture assays, KIF20A-overexpressing HCC cells suppressed GzMB and IFN-γ production in CD8^+^ T cells, an effect reversed by concurrent c-Myc knockdown (Figures 5H and 5I). Furthermore, c-Myc ablation significantly impaired the colony-forming capacity of KIF20A-overexpressing cells when co-cultured with CTLs (Figures 5J and 5K).

In summary, KIF20A promotes immune evasion of HCC cells by regulating PD-L1 expression via c-Myc.

### Inhibiting glycolysis markedly improves the therapeutic effectiveness of PD-1 inhibitors in preclinical HCC models

PD-1 inhibitor-based immunotherapy has demonstrated encouraging results in treating advanced HCC. However, the suboptimal therapeutic efficacy due to high interpatient heterogeneity highlights the necessity of developing rational combination therapy strategies. We investigated the antitumor efficacy and molecular mechanisms of oxamate combined with an anti-PD-1 inhibitor in a preclinical HCC model.

**Figure 6:**
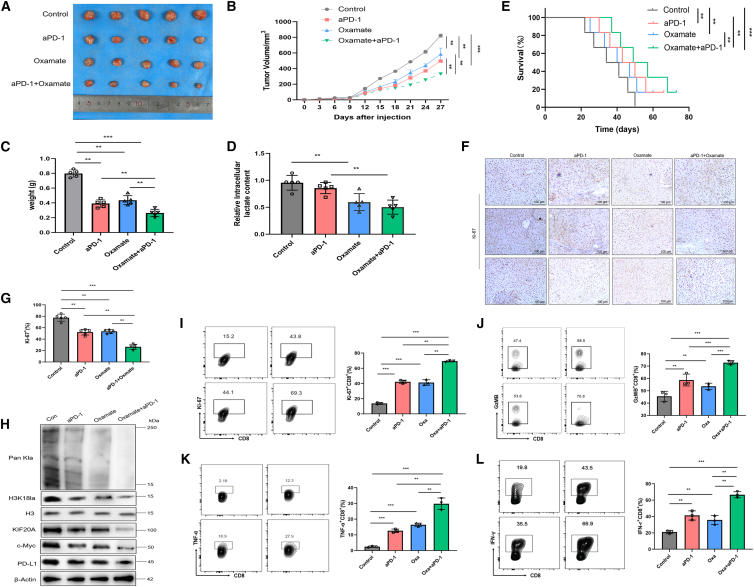
Glycolysis inhibition significantly enhances the efficacy of PD-1 inhibitors in preclinical HCC models 2×10^5^ Hepa1-6 cells in the logarithmic growth phase were implanted subcutaneously into C57BL/6J mice. When tumor volumes reached 100 mm^3^, mice were randomly divided into four groups. Representative images of tumor sizes (A), tumor volumes (B), tumor weights (C), and intratumoral lactate contents (D) in each group (*n* = 6). (E) Survival curves of mice in different treatment groups (*n* = 6). (F and G) Ki-67 IHC results of tumors in different treatment groups (*n* = 5), scale bars, 100 μm. (H) WB analysis of protein levels of Pan Kla, H3K18la, KIF20A, c-Myc, and PD-L1 in tumors (*n* = 5). FC analysis of the proportions of intratumoral Ki-67^+^ (I), GzMB^+^ (J), TNF-α^+^ (K), IFN-γ^+^, and (L) CTLs with representative scatterplots (*n* = 5). Cell aggregates and debris were first excluded, followed by gating on the target cell populations and markers of interest. Data are presented as mean ± SD. (B) was analyzed by two-way ANOVA, and (C, D, F, and I–L) were analyzed by one-way ANOVA. ∗∗*p* < 0.01 and ∗∗∗*p* < 0.001. Experiments were repeated three times.

Mice bearing subcutaneous Hepa1-6 tumors (right dorsal flank) were randomized to four groups: control, PD-1 inhibitor, oxamate, and combination (oxamate + PD-1 inhibitor). The findings indicated that single-agent therapy with either a PD-1 inhibitor or oxamate had only modest inhibitory effects on tumor growth. Conversely, the combination of oxamate and anti-PD-1 substantially retarded tumor progression (Figure 6A), resulting in significantly smaller tumor volumes and weights compared to monotherapy groups (Figures 6B and 6C), along with a notable reduction in intratumoral lactate levels (Figure 6D), suggesting a synergistic effect in suppressing HCC development. Notably, combination therapy conferred a significant survival advantage over single-agent treatments in the murine model (Figure 6E). Additionally, IHC assessment of Ki-67 expression in HCC tissues showed a marked decrease in the combination group relative to monotherapy groups (Figures 6F and 6G), along with lowered expression of Pan Kla, H3K18la, KIF20A, c-Myc, and PD-L1 (Figure 6H). FC analysis further revealed that combined treatment with oxamate and PD-1 inhibitor synergistically enhanced Ki-67^+^ proliferative CTLs (Figure 6I), GzMB expression (Figure 6J), TNF-α (Figure 6K), and IFN-γ (Figure 6L) levels in CTLs.

These results lead us to conclude that glycolysis blockade significantly enhances the efficacy of PD-1 inhibitors by potentiating the antitumor response of CTLs.

### Depletion of KIF20A reverses lactic acid-induced progression and immunosuppression of HCC

In this study, to further confirm the functional necessity of KIF20A in mediating lactate-induced HCC immune evasion and tumor progression via the lactate-H3K18la-KIF20A-*c*-Myc-PD-L1 axis, we employed an orthotopic xenograft tumor model of HCC, which closely mimics the *in vivo* microenvironment and pathological characteristics of human HCC. This model was selected to better reflect the actual regulatory role of KIF20A in tumor progression and immunosuppression under physiological conditions, as orthotopic tumors can more accurately recapitulate the interaction between tumor cells and the surrounding immune microenvironment compared with subcutaneous xenografts.

**Figure 7:**
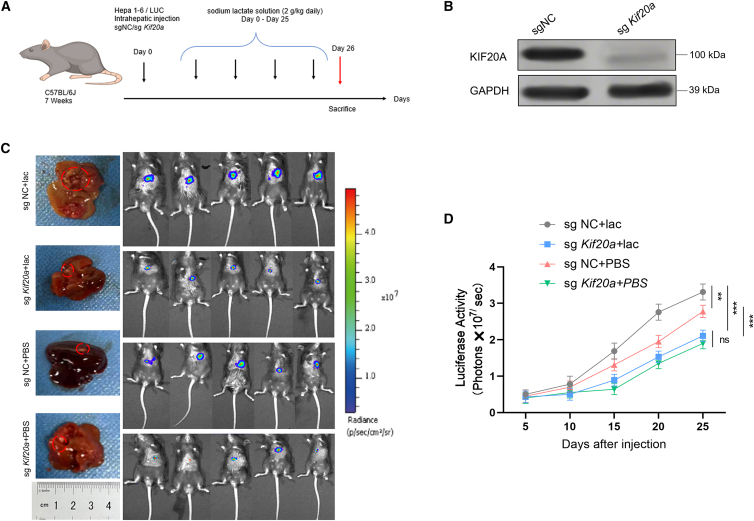
Depletion of KIF20A reverses lactic acid-induced progression of HCC (A and B) Experimental design for orthotropic HCC mouse model. C57BL/6 mice were inoculated with Hepa1-6/LUC-sgNC (*n* = 8) or Hepa1-6-sg *Kif20a* (*n* = 8), followed by daily intraperitoneal injection of lactate (2 g/kg), and tissues were harvested at 25 days post-inoculation. (C and D) Representative gross images of liver tumors in the designated groups, along with non-invasive bioluminescent imaging of tumors in mice and the quantitative analysis (*n* = 6). Results are presented as mean ± SD. ∗∗*p* < 0.01 and ∗∗∗*p* < 0.001. Experiments were repeated three times.

The detailed experimental workflow, including the establishment of orthotopic HCC models, lentiviral-mediated sg *Kif20a* knockout in HCC cells, verification of KO efficiency, and *in vivo* monitoring of tumor growth, is presented in Figure 7A. To ensure the reliability of subsequent experiments, we first validated the KIF20A knockout efficiency at both the mRNA and protein levels using qPCR and WB analysis, respectively. The results (Figure 7B) clearly demonstrated that KIF20A expression was significantly downregulated in the sg *Kif20a* group compared with the control group, confirming the successful construction of KIF20A-deficient HCC cells and stable orthotopic tumor models.

Tumor growth dynamics were non-invasively monitored at regular intervals via *in vivo* bioluminescence imaging, which allows for real-time, quantitative assessment of tumor burden in living mice. At 25 days post-tumor implantation, the final tumor sizes of each designated group (control group, lactate-treated group, lactate + sg *Kif20a* group, and sg *Kif20a* group alone) were measured and recorded, and the BLI imaging data were quantitatively analyzed using professional imaging software. As shown in Figures 7C and 7D, the lactate-treated group exhibited significantly larger tumor sizes and higher bioluminescence intensity compared with the control group, indicating that lactate effectively promotes HCC tumor growth *in vivo*. In contrast, the depletion of KIF20A significantly reduced tumor size and bioluminescence signal in the lactate-treated group, which was comparable to the control group, suggesting that KIF20A depletion can effectively reverse lactate-induced HCC progression.

## Discussion

The mechanisms of immune evasion in HCC are a key research focus and challenge in tumor immunology. Here, we uncover a pathway: H3K18 lactylation drives HCC immune escape via the KIF20A/c-Myc/PD-L1 axis, and combined glycolysis and PD-1 inhibitors markedly boost anti-HCC efficacy. These insights offer molecular mechanisms of HCC immune evasion and experimental support for developing combination therapies.

The TME constitutes a distinctive and intricate ecosystem arising from dynamic interactions between tumor and host cells during carcinogenesis. In this microenvironment, cancer cells display heightened glycolytic activity, resulting in excessive lactate accumulation, a metabolic hallmark that critically contributes to the formation of an immunosuppressive milieu, thereby directly obstructing the activation and execution of anti-tumor immunity.^28^ Specifically, the immunosuppressive effects of lactate are multifaceted: via monocarboxylate transporter 1-mediated influx, lactate enters tumor-infiltrating regulatory T cells, supporting their proliferation and suppressive functions, ultimately shortening the survival of tumor-bearing mice^29^; concurrently, lactate reduces nicotinamide adenine dinucleotide levels in T cells, inducing their apoptosis.^30^ Furthermore, it binds to G protein-coupled receptor 132 to promote M2-like macrophage polarization, indirectly facilitating tumor cell migration and invasion.^31^ We confirm that inhibiting glycolysis in HCC reactivates CTL cytotoxicity, effectively curbing tumor growth. Mechanistically, this reinforces lactate’s immunosuppressive role. Conceptually, elucidating the direct impact of lactate and histone lactylation on CD8^+^ T cells will offer a crucial framework for linking metabolic reprogramming to immune evasion.

Notably, H3K18la exerts multifaceted pro-tumor effects in HCC beyond immune evasion: It is upregulated by sublethal heat stress via glycolysis, binds the NSF1 promoter to inhibit ferroptosis and promote metastasis,^20^ and specifically regulates immune checkpoint-related pathways, differing functionally from H3K9la and H3K14la as a unique epigenetic marker for HCC immune evasion,^32^ underscoring its specificity in HCC pathogenesis and potential as a multi-target therapeutic candidate. Lactate in the body is primarily derived from glycolytic metabolites. As a metabolic substance, lactate plays important roles in energy metabolism, cell signaling, and immune regulation.^33^^,^^34^^,^^35^^,^^36^^,^^37^ Histone lactylation is an epigenetic mark generated through the lactate-dependent modification of histone lysine residues. Aberrant regulation of this lactylation process disturbs transcriptional homeostasis and promotes pathological conditions, including malignancies.^38^^,^^39^ Recently, in digestive system diseases, Li et al. demonstrated that lactate-driven epigenetic modifications create a molecularly distinct subpopulation via the LDHA-NDRG1 axis, enabling senescence resistance. This finding provides mechanistic insights into the heterogeneity of HCC.^40^ Emerging evidence reveals multifaceted roles of lactylation in tumor progression: It sustains pancreatic cancer survival during glucose deprivation by elevating NMNAT1 lactylation to maintain nuclear NAD^+^ synthesis,^41^ and concurrently promotes HCC metastasis by facilitating tumor exosome secretion via Rab7A lactylation.^42^ These findings significantly expand the mechanistic understanding of lactylation in oncology.

These emerging findings advance our understanding of the molecular mechanisms of tumor immune evasion. This study not only confirms that H3K18 lactylation binds to the KIF20A promoter to promote KIF20A transcription, and KIF20A further enhances PD-L1 expression by regulating c-Myc, ultimately driving HCC immune evasion, but also identifies KIF20A as a downstream target gene of H3K18la. As a key core node in the lactate-H3K18la pathway regulating the immune microenvironment, KIF20A enables this finding to further deepen our understanding of the mechanism by which H3K18la mediates the interaction between tumor cells and the TME.

Immune checkpoint inhibitors have revolutionized cancer treatment and gained approval for multiple cancer types, yet monotherapy often fails to fully arrest tumor progression. To address this, researchers have recently explored combining metabolic regulation with immunotherapy to enhance efficacy.^43^ Studies show that targeting tumor glycolysis can augment immune responses through multiple pathways: Disrupting glucose-induced NSUN2 activation promotes CTL infiltration via the cGAS/STING pathway, reversing anti-PD-L1 resistance.^44^ In melanoma, combined inhibition of PD-1 and glycolytic pathways blocks tumor cell LDHA activity, activating synergistic cytotoxicity of natural killer cells and CTLs.^45^ In HCC, targeting glycolysis inhibits the lactylation of moesin at Lys72, perturbing Treg stability and immunosuppressive function, thereby enhancing anti-PD-1 efficacy.^46^ Moreover, combining glycolysis regulation with other ICIs shows promise: Blocking CTLA-4 induces Treg instability by limiting their glycolytic capacity, significantly improving outcomes in glycolysis-deficient tumor-bearing mice.^47^ In gastric cancer models, targeting hexokinase 2 in combination with PD-L1 inhibitors enhances T cell cytotoxicity by reducing intratumoral glucose consumption.^48^ Luo et al.^49^ Recently developed a TME-responsive bispecific aptamer nanocomplex, which dissociates in the acidic TME to release aptPDL1, aptCTLA-4, and BAY-876, the latter inhibits glycolysis to reduce PD-L1 glycosylation, enhancing immune checkpoint blockade, while regulating Treg metabolic phenotypes, synergizing with dual immune checkpoint blockade to effectively suppress triple-negative breast cancer growth and offering a strategy for clinical immunotherapy. These findings suggest that reducing tumor glucose dependence may represent a common mechanism to enhance immune checkpoint blockade efficacy, providing broader theoretical support and application prospects for combining glycolysis inhibitors with immunotherapy.

KIF20A acts as a key tumor-associated antigen in immunotherapy, closely linked to immune response regulation and immunotherapeutic strategies such as peptide vaccines. Studies have shown that KIF20A-derived peptides can bind MHC-I molecules such as HLA-A2 and HLA-A2402, activating CTLs to specifically kill KIF20A-expressing tumor cells.^50^^,^^51^ Additionally, long peptides can simultaneously activate CD4^+^ helper T cells and CD8^+^ CTLs, enhancing the comprehensiveness and durability of immune responses.^52^ In pancreatic cancer, phase I/II trials of the KIF20A-66 peptide vaccine reported a 72% disease stabilization rate and notably extended patient survival.^53^ Peptide cocktail vaccines containing KIF20A induce specific T cell responses in patients with advanced pancreatic cancer and biliary tract cancer, stabilizing disease and extending PFS.^54^ In postoperative adjuvant vaccine therapy, KIF20A-specific CD8^+^ T cell responses correlate with longer DFS in patients with pancreatic cancer.^55^ Collectively, these studies confirm that KIF20A-targeting vaccines can be combined with ICIs and chemotherapy to enhance T cell infiltration and activity, improve the immunosuppressive TME, and boost therapeutic efficacy. This study further expands the functional boundaries of KIF20A; it not only confirms that KIF20A is a direct target of H3K18la but also clarifies its non-canonical mechanism of mediating immune evasion through the c-Myc/PD-L1 axis.

### Limitations of the study

While we show that EP300 binds the KIF20A promoter with H3K18la in a glycolysis-sensitive manner, key questions persist. These include the potential crosstalk between H3K18la and EP300-driven acetylation, and the identification of functional lactylation sites on KIF20A via targeted proteomics/mutagenesis, which are essential for a complete pathway delineation.

## Resource availability

### Lead contact

Further information and requests for resources and reagents should be directed to and will be fulfilled by the Lead Contact, Jia Li (jiali_0503@tmu.edu.cn).

### Materials availability

This study did not generate new animals, cell lines, or unique reagents.

### Data and code availability

• Data presented in this study are available from the corresponding author upon reasonable request.
• This article does not report the original code.
• Any additional information required to reanalyze the data reported in this article is available from the lead contact upon request.

## Acknowledgments

This study was jointly supported by the Tianjin Key Medical Discipline Construction Project (TJYXZDXK-3-019B), the Integrated Traditional Chinese and Western Medicine Discipline Enhancement Program of 10.13039/501100010104Tianjin Medical University (2024XKZXY09), and the Hengrui Hebei Innovative Development Medical Cooperation Program of The Third Hospital of Hebei Medical University (HR202502033).

## Author contributions

Shujia Chen conceived and designed the main research, performed experiments, and drafted the manuscript. Z. Q. provided support for the project’s implementation in terms of instrumentation and equipment, as well as in the design of plans and guidance for planning. L. Z. and T. M. provided support for experimental instruments and collected samples. P. H. and J. L. contributed to part of the experimental implementation and data statistics. Jiancun Hou is mainly responsible for the collection of clinical tissue specimens. F. W. and J. L. supervised the experimental implementation and revised the manuscript.

## Declaration of interests

The authors declare no competing interests.

## STAR★Methods

### Key resources table

**Table:** 

REAGENT or RESOURCE	SOURCE	IDENTIFIER
**Antibodies**		

KIF20A Polyclonal antibody	Proteintech	Cat# 15911-1-AP; RRID: AB_3740177
c-Myc Polyclonal antibody	Proteintech	Cat# 10828-1-AP; RRID: AB_2148585
PD-L1/CD274 Polyclonal antibody	Proteintech	Cat# 28076-1-AP; RRID: AB_10597552
LDHA-Specific Polyclonal antibody	Proteintech	Cat# 19987-1-AP; RRID: AB_10646429
LDHB Polyclonal antibody	Proteintech	Cat# 14824-1-AP; RRID: AB_10700003
Beta Actin Polyclonal antibody	Proteintech	Cat# 20536-1-AP; RRID: AB_10700003
Anti-L-Lactyl Lysine Rabbit mAb	PTM BIO	Cat# PTM-1401; RRID: AB_2942013
Anti-L-Lactyl-Histone H3 (Lys18) Rabbit mAb	PTM BIO	Cat# PTM-1406RM; RRID: AB_2909438
Anti-L-Lactyl-Histone H3 (Lys18) Rabbit mAb-ChIP Grade	PTM BIO	Cat# PTM-1427RM; RRID: AB_3076698
Histone H3K18ac antibody (pAb)	Proteintech	Cat# 39130; RRID: AB_3740186
Histone H3 Polyclonal antibody	Proteintech	Cat# 17168-1-AP; RRID: AB_2716755
Granzyme B Monoclonal Antibody, PE	Thermo Fisher	Cat# 12-8898-82; RRID: AB_3740188
Anti-human/mouse Granzyme B-APC/Cy7	BioLegend	Cat# 372227; RRID: AB_2936711
PE Anti-Mouse IFN-γ Antibody	Elabscience	Cat# E-AB-F1101D; RRID: AB_3740190
APC Anti-Mouse IFN-γ Antibody	Elabscience	Cat# E-AB-F1101E; RRID: AB_3740191
FITC Anti-Mouse IFN-γ Antibody	Elabscience	Cat# E-AB-F1101C; RRID: AB_3661692
Ki-67 Polyclonal antibody	Proteintech	Cat# 27309-1-AP; RRID: AB_3740193
p300 (D8Z4E) Rabbit mAb	CST	Cat# 86377; RRID: AB_3740194
InVivoMAb anti-mouse PD-1	Bio X Cell	Cat# BE0273; RRID: AB_3740195
InVivoMAb anti-human CD3	Bio X Cell	Cat# BE0001-2; RRID: AB_3740196
InVivoMAb anti-human CD28	Bio X Cell	Cat# BE0248; RRID: AB_3740197
InVivoMAb rat IgG2a	Bio X Cell	Cat# BE0089; RRID: AB_3740198
InVivoMAb anti-mouse CD8α	Bio X Cell	Cat# BE0117; RRID: AB_3740199
FITC Anti-Mouse CD3 Antibody	Elabscience	Cat# E-AB-F1013C; RRID: AB_3065041
APC Anti-Mouse CD8a Antibody	Elabscience	Cat# E-AB-F1104E; RRID: AB_3740201
PerCP Anti-Mouse CD45 Antibody	Elabscience	Cat# E-AB-F1136F; RRID: AB_3740202
Anti-Human CD44 Antibody	Elabscience	Cat# E-AB-F1215A; RRID: AB_3740203
APC Anti-Mouse CD62L Antibody	Elabscience	Cat# E-AB-F1011E; RRID: AB_3740204
PE/Cyanine7 Anti-Mouse TNFα Antibody	Elabscience	Cat# AN00567H; RRID: AB_3740205
FITC Anti-Human Ki-67 Antibody	Elabscience	Cat# AN00540C; RRID: AB_3740206

**Chemicals, peptides, and recombinant proteins**		

DMEM	GIBCO	Cat# 11965-092
RPMI-1640	Thermo Fisher	Cat# 17104019
Fetal Bovine Serum	GIBCO	Cat# 10437-028
Penicillin-Streptomycin	GIBCO	Cat# 15140-122
Lipofectamine 2000	Yeasen	Cat# 40802ES01
puromycin	Solarbio	Cat# P8230
formaldehyde	MACKLIN	Cat# 50-00-0
ChIP lysis buffer	Beyotime	Cat# P2078
RNase A	Beyotime	Cat# ST579
Proteinase K	Beyotime	Cat# ST535
2-DG	APExBIO	Cat# B1027
oxamate	MCE	Cat# HY-W013032A
DNase I	Roche	Cat# 10104159001 Roche
Lactate detection kit	Solarbio	Cat# BC2235
CD8a^+^ T Cell Isolation Kit	Miltenyi Biotec	Cat# 130-104-075
recombinant human IL-2	PeproTech	Cat# 200-02
crystal violet	Servicebio	Cat# G1014
Percoll gradient centrifugation	Cytiva	Cat# 17-0891-01
pMD2.G plasmid	Addgene	Cat# 12259
psPAX2 plasmid	Addgene	Cat# 12260

**Experimental models: Cell lines**		

Huh7	FUHENG BIOLOGY	Cat# FH0075
HCCLM3	FUHENG BIOLOGY	Cat# FH0096
MHCC97-H	FUHENG BIOLOGY	Cat# FH0095
MHCC97-L	FUHENG BIOLOGY	Cat# FH0097
Hep3B	FUHENG BIOLOGY	Cat# FH0861
Hepa 1-6	FUHENG BIOLOGY	Cat# FH0862
HEK293T	FUHENG BIOLOGY	Cat# FH0952

**Experimental models: Organisms/strains**		

Mouse: C57BL/6J	Beijing HFK Bio-Technology.co., LTD	Stock No. 0055670
Mouse: Liver-specific KIF20A knockout C57BL/6J	cyagen (Nanjing, China)	Stock No. S-KO-17831

**Oligonucleotides**		

Primer sequences for qPCR	Table S2	–

**Software and algorithms**		

Prism 9.0	GraphPad	https://www.graphpad.com
ImageJ	ImageJ	https://imagej.nih.gov/ij
FlowJov.10.5.3	Treestar	https://www.flowjo.com/solutions/flowjo/downloads
Illustrator	Adobe	https://www.adobe.com

### Experimental model and study participant details

#### Clinical sample

This study included surgical specimens from 89 patients with hepatocellular carcinoma (HCC), consisting of 89 pairs of HCC tissues and their adjacent non-tumor tissues, which were designated as the experimental group and the control group, respectively. All specimens were verified by pathological examination. After collection, all tissues were stored at -80°C for future use. All procedures were approved by the Ethics Committee of Tianjin Second People's Hospital (Approval No. LL-BG-032), and written informed consent was obtained from each participant. This study was conducted in accordance with the ethical principles outlined in the Declaration of Helsinki.

#### Cell culture

Cell lines, including human HCC cell lines (Huh7, HCCLM3, MHCC97-H, MHCC97-L, Hep3B, Hepa 1-6), mouse Hepa 1-6 and normal hepatocyte AML12, were all obtained from the Cell Bank of the Chinese Academy of Sciences (all authenticated by STR analysis). These cell lines were cultured in DMEM medium supplemented with 10% fetal bovine serum and 1% penicillin-streptomycin, and incubated at 37°C in a humidified atmosphere containing 5% CO_2_. All cell lines were routinely tested negative for mycoplasma contamination with a mycoplasma assay kit (Yeasen, Shanghai, China).

#### Mice

Six-week-old male C57BL/6 mice were obtained from Beijing Huafukang Biotechnology Co., Ltd. All mice were housed in a specific pathogen-free (SPF) facility at the Institute of Radiation Medicine, Chinese Academy of Medical Sciences. All animal experiments were approved by the Ethics Committee of the Institute of Radiation Medicine, Chinese Academy of Medical Sciences (Approval No.: IRM/2-IACUC-2311-006).

### Method details

#### Transfection and lentiviral infection

Huh7 and HCCLM3 cells were transfected with siRNAs (LDHA, LDHB, KIF20A, or c-Myc) or KIF20A overexpression plasmids using Lipofectamine 2000. Transfection efficiency was verified 48 h later. LDHA/LDHB double knockout (DKD) cell lines were constructed by transducing lentiviral particles containing LDHA and LDHB shRNAs or transfecting pcDNA3.1 plasmids encoding KIF20A, with positive clones selected using puromycin.

sgRNA design was conducted by Santa Cruz, with sequences listed in Table S2. The full-length open reading frame of mouse KIF20A (NM_005733.3) was cloned into the CMV-MCS-3XFlag-PGK-Puro lentiviral vector. For lentivirus preparation, HEK293T cells were cotransfected with a mixture comprising 8 μg target plasmid DNA, 2 μg pMD2.G plasmid, 6 μg psPAX2 plasmid, and 36 μl Lipofectamine 2000. Culture medium was refreshed 6 h post-transfection, and cell supernatants were harvested at 48 and 72 h post-transfection for downstream experiments.

#### Luciferase reporter assay

The KIF20A promoter fragment was PCR-amplified and cloned into the pGL3-basic vector to generate the recombinant reporter plasmid PGL3-KIF20A. The pGL3-PD-L1 plasmid was constructed following the same method. Huh7 and HCCLM3 cells were seeded in 24-well plates to 80% confluency, then co-transfected with PGL3-KIF20A (or pGL3-PD-L1) and the pRL-TK reference plasmid; the control group used the empty pGL3-basic vector. To assess KIF20A promoter activity, transfected cells were maintained in culture medium supplemented with or without lactate for 24 hours. For evaluating PD-L1 promoter regulation, cells underwent co-transfection with pGL3-PD-L1, pRL-TK, KIF20A overexpression construct, and c-Myc siRNA, subsequently cultured for 48 hours. The assay was measured via the DLR™.

#### Chromatin immunoprecipitation (ChIP)-PCR

HCC cells in logarithmic growth phase were fixed using 1% formaldehyde for 10 minutes at ambient temperature, followed by reaction quenching with glycine. After collection, cells were subjected to lysis in chilled ChIP lysis buffer on ice for 30 minutes, and cellular lysates were subsequently harvested. Chromatin fragmentation to 200-500 bp segments was achieved through sonication, with supernatants collected following centrifugation. An aliquot was preserved as an input control, while the remaining lysate was subjected to overnight rotation at 4°C with 2 μg of either anti-H3K18la, anti-EP300, anti-c-Myc antibodies, or IgG control. Protein A/G agarose beads were introduced and incubated at 4°C for 2 hours. Immune complexes underwent three washing cycles with the designated buffer, followed by a single wash with TE buffer, then eluted at 65°C for 30 minutes. Cross-link reversal and protein digestion were performed using RNase A and Proteinase K at 65°C for 4 hours. Quantitative PCR analysis was executed on a Bio-Rad system employing specific primers, and promoter enrichment values were normalized against input controls.

#### Lactate level measurement

For *in vitro* assays, Huh7 cells were treated with solvent control, 2-DG, or oxamate for 24 h. Cell lysates were centrifuged, and supernatants were collected to measure intracellular lactate concentration. Similarly, Huh7 cells transfected with siLDHA, siLDHB, or both were seeded in culture dishes until 80%-90% confluent, cultured for 24 h, and lactate levels in supernatants of lysates were measured. For tumor tissue analysis *in vivo*, samples were mechanically disrupted in extraction buffer at a 1:10 (w/v) ratio, followed by centrifugation to obtain the supernatant fraction. Lactate levels were quantified employing a commercial lactate detection kit, with subsequent normalization performed relative to either total protein content or tissue mass.

#### Immunohistochemistry (IHC)

Formalin-fixed paraffin-embedded tissue sections underwent IHC staining. Following antigen retrieval and peroxidase inhibition, the sections were incubated sequentially with primary antibodies (Pan Kla, H3K18la, KIF20A, Ki-67) at 4°C overnight, followed by incubation with secondary antibodies. DAB was used for visualization. Staining was quantitatively assessed by two pathologists using the IRS.

#### *In vitro* T cell co-culture assay

CD8^+^ T cells were purified from PBMCs with a CD8a^+^ T Cell Isolation Kit per the protocol. The procedure involved first incubating PBMCs with a biotin-labeled antibody mixture and anti-biotin-coated magnetic beads at 4°C, followed by positive selection through the MidiMACS™ separation system. Isolated cells were pre-activated in medium containing 5 μg/ml CD3/CD28 stimulating antibodies for 3 days, then cultured with 20 ng/ml recombinant human IL-2 for 4 days to complete activation. Activated CD8^+^ T cells were co-cultured with Huh7 cells at a 1:3 ratio, with 1 μg/ml CD3/CD28 antibodies added to maintain stimulation. Functional analyses of co-cultures included: (1) tumor colony formation (quantified by crystal violet staining); (2) detection of effector molecules in CD8^+^ T cells (GzMB, IFN-γ); (3) dynamic monitoring of tumor proliferation.

#### Isolation of tumor-infiltrating leukocytes (TILs)

Fresh tumor tissues were washed with ice-cold PBS to eliminate blood and necrotic tissue, then cut into 1-2 mm^3^ pieces in a sterile Petri dish. Fragments were transferred to centrifuge tubes containing digestion buffer (RPMI-1640 medium with 200 μg/ml collagenase IV and 40 μg/ml DNase I and enzymatically dissociated at 37°C with shaking for 1.5 h, with gentle pipetting every 30 min to enhance digestion. Digested cell suspensions were filtered through a 70 μm cell strainer, and filtrates were centrifuged at 1500 rpm for 5 min. Pellets were resuspended in PBS and centrifuged again. TILs were isolated using Percoll gradient centrifugation: diluted Percoll was used to prepare discontinuous density gradients, and cell suspensions were layered on top. Following a 20-minute centrifugation at 800 × g, the buffy coat layer containing tumor-infiltrating lymphocytes was carefully isolated. This cellular fraction underwent two PBS washing steps before being reconstituted in complete culture medium for further experimental procedures.

#### Flow cytometry (FC)

TILs or CD8^+^ T cells from co-cultures were stained for surface markers (CD45, CD3, CD8, CD44, CD62L) and intracellular molecules (GzMB, IFN-γ), then analyzed using a BD FACSVerse system. Data were analyzed using FlowJo to determine the proportion of positive cells.

#### Animal models

Animal experiments were carried out in compliance with the approval of the Animal Ethics Committee of Tianjin Medical University. Male C57BL/6J mice (6-8 weeks old, weighing 15-20 g) were obtained from Beijing Huafukang Biotechnology Co., Ltd., and housed in SPF environments.

#### Subcutaneous tumor model

C57BL/6J mice received subcutaneous injections of 2×10^5^ control or LDHA/B DKO Hepa 1-6 cells (in 150 μl PBS) into the right dorsal flank. Tumor volume was calculated every 3 days using the formula V = ab^2^/2 (a, major axis; b, minor axis), and body weight and survival were monitored. Combination therapy: When tumors reached 100 mm^3^, mice were randomized to four groups: (1) control (isotype IgG); (2) oxamate (250 mg/kg/day, intraperitoneal); (3) anti-PD-1mAb (250 μg/mouse, intraperitoneal every 3 days); (4) combination (anti-PD-1 + oxamate).

##### CD8^+^ T lymphocyte elimination

Animals received intraperitoneal administration of anti-CD8 monoclonal antibody (500 μg per mouse) 48 hours before implantation with 2×10^5^ Hepa 1-6 cells, with subsequent biweekly injections to sustain lymphocyte depletion.

##### Orthotopic xenograft HCC model

Hepa 1-6-Luc cells (CatNo. CL-1007, Procell) were used for *in vivo* imaging. Mice were induced into anesthesia using 2% isoflurane, followed by making a 0.5 cm abdominal incision. Subsequently, a total of 2×10^5^ Hepa 1-6 (KIF20A-KO-sg) cells, suspended in 10 μl of sterile PBS, were injected into the left liver lobe. The incision was then sutured, and the mice were permitted to recover. The mice were randomly assigned into four groups (n = 8): (1) sgNC + lactate; (2) sg *Kif20a* + lactate; (3) sgNC + PBS; (4) sg *Kif20a* + PBS. The dosage and concentration of sodium lactate and PBS were consistent with those used in the primary HCC model. Tumor volumes were measured every 5 days, and *in vivo* imaging was performed on days 5, 10, 15, 20, and 25 to observe and record tumor sizes. On day 26, the mice were euthanized via cervical dislocation, and the tumor tissues were harvested for subsequent experiments.

#### Statistical analysis

Statistical analyses were performed with GraphPad Prism 9. All data are presented and plotted as the mean ± standard deviation (SD), as shown in the figures and results. A p-value < 0.05 was considered statistically significant. Differences were considered statistically significant when *P* < 0.05 (two-tailed, ∗*P* < 0.05, ∗∗*P* < 0.01, ∗∗∗*P* < 0.001). Each experiment was performed in triplicate.

## References

[1] Foerster F., Gairing S.J., Müller L., Galle P.R. (2022). NAFLD-driven HCC: Safety and efficacy of current and emerging treatment options. J. Hepatol..

[2] Zheng J., Wang S., Xia L., Sun Z., Chan K.M., Bernards R., Qin W., Chen J., Xia Q., Jin H. (2025). Hepatocellular carcinoma: signaling pathways and therapeutic advances. Signal Transduct. Target. Ther..

[3] Lu J., O D.C., Tsui Y.M., Ng I.O. (2025). Cancer stem cells in hepatocellular carcinoma: platforms, updates, challenges and future perspectives. Cancer Biol. Med..

[4] Zhu C.-X., Yan K., Chen L., Huang R.R., Bian Z.H., Wei H.R., Gu X.M., Zhao Y.Y., Liu M.C., Suo C.X. (2024). Targeting OXCT1-mediated ketone metabolism reprograms macrophages to promote antitumor immunity via CD8+ T cells in hepatocellular carcinoma. J. Hepatol..

[5] Liu F., Chen H., Wu S., Zhu C., Zhang M., Rui W., Zhou D., Wang Y., Lin X., Zhao X., Ye Y. (2025). Neoepitope BTLA(P267L)-specific TCR-T cell immunotherapy unlocks precision treatment for hepatocellular carcinoma. Cancer Biol. Med..

[6] Yao C., Wu S., Kong J., Sun Y., Bai Y., Zhu R., Li Z., Sun W., Zheng L. (2023). Angiogenesis in hepatocellular carcinoma: mechanisms and anti-angiogenic therapies. Cancer Biol. Med..

[7] Apostolova P., Pearce E.L. (2022). Lactic acid and lactate: revisiting the physiological roles in the tumor microenvironment. Trends Immunol..

[8] Certo M., Llibre A., Lee W., Mauro C. (2022). Understanding lactate sensing and signalling. Trends Endocrinol. Metab..

[9] Yu J., Chai P., Xie M., Ge S., Ruan J., Fan X., Jia R. (2021). Histone lactylation drives oncogenesis by facilitating m6A reader protein YTHDF2 expression in ocular melanoma. Genome Biol..

[10] Jiang J., Huang D., Jiang Y., Hou J., Tian M., Li J., Sun L., Zhang Y., Zhang T., Li Z. (2021). Lactate Modulates Cellular Metabolism Through Histone Lactylation-Mediated Gene Expression in Non-Small Cell Lung Cancer. Front. Oncol..

[11] Hua G., Liu Y., Li X., Xu P., Luo Y. (2014). Targeting glucose metabolism in chondrosarcoma cells enhances the sensitivity to doxorubicin through the inhibition of lactate dehydrogenase-A. Oncol. Rep..

[12] Peng T., Sun F., Yang J.-C., Cai M.H., Huai M.X., Pan J.X., Zhang F.Y., Xu L.M. (2024). Novel lactylation-related signature to predict prognosis for pancreatic adenocarcinoma. World J. Gastroenterol..

[13] From the American Association of Neurological Surgeons AANS American Society of Neuroradiology ASNR Cardiovascular and Interventional Radiology Society of Europe CIRSE Canadian Interventional Radiology Association CIRA Congress of Neurological Surgeons CNS European Society of Minimally Invasive Neurological Therapy ESMINT European Society of Neuroradiology ESNR European Stroke Organization ESO Society for Cardiovascular Angiography and Interventions SCAI Society of Interventional Radiology SIR Society of NeuroInterventional Surgery SNIS and World Stroke Organization WSO, Sacks D., Baxter B., Campbell B.C.V., Carpenter J.S., Cognard C., Dippel D., Eesa M., Fischer U., Hausegger K. (2018). Multisociety Consensus Quality Improvement Revised Consensus Statement for Endovascular Therapy of Acute Ischemic Stroke. Int. J. Stroke.

[14] Chai P., Zhao F., Jia R., Zhou X., Fan X. (2025). Lactate/lactylation in ocular development and diseases. Trends Mol. Med..

[15] Yang J., Luo L., Zhao C., Li X., Wang Z., Zeng Z., Yang X., Zheng X., Jie H., Kang L. (2022). A Positive Feedback Loop between Inactive VHL-Triggered Histone Lactylation and PDGFRβ Signaling Drives Clear Cell Renal Cell Carcinoma Progression. Int. J. Biol. Sci..

[16] Wang G., Zou X., Chen Q., Nong W., Miao W., Luo H., Qu S. (2024). The relationship and clinical significance of lactylation modification in digestive system tumors. Cancer Cell Int..

[17] Xiong J., He J., Zhu J., Pan J., Liao W., Ye H., Wang H., Song Y., Du Y., Cui B. (2022). Lactylation-driven METTL3-mediated RNA m6A modification promotes immunosuppression of tumor-infiltrating myeloid cells. Mol. Cell.

[18] Yang Z., Yan C., Ma J., Peng P., Ren X., Cai S., Shen X., Wu Y., Zhang S., Wang X. (2023). Lactylome analysis suggests lactylation-dependent mechanisms of metabolic adaptation in hepatocellular carcinoma. Nat. Metab..

[19] Li W., Zhou C., Yu L., Hou Z., Liu H., Kong L., Xu Y., He J., Lan J., Ou Q. (2024). Tumor-derived lactate promotes resistance to bevacizumab treatment by facilitating autophagy enhancer protein RUBCNL expression through histone H3 lysine 18 lactylation (H3K18la) in colorectal cancer. Autophagy.

[20] Huang J., Xie H., Li J., Huang X., Cai Y., Yang R., Yang D., Bao W., Zhou Y., Li T., Lu Q. (2025). Histone lactylation drives liver cancer metastasis by facilitating NSF1-mediated ferroptosis resistance after microwave ablation. Redox Biol..

[21] Wei J., Chen X., Li Y., Li R., Bao K., Liao L., Xie Y., Yang T., Zhu J., Mao F. (2022). Cucurbitacin B-induced G2/M cell cycle arrest of conjunctival melanoma cells mediated by GRP78-FOXM1-KIF20A pathway. Acta Pharm. Sin. B.

[22] Yang M., Huang H., Zhang Y., Wang Y., Zhao J., Lee P., Ma Y., Qu S. (2024). Identification and validation of KIF20A for predicting prognosis and treatment outcomes in patients with breast cancer. Sci. Rep..

[23] Meng X., Li W., Yuan H., Dong W., Xiao W., Zhang X. (2022). KDELR2-KIF20A axis facilitates bladder cancer growth and metastasis by enhancing Golgi-mediated secretion. Biol. Proced. Online.

[24] You Z., Lei Y., Yang Y., Zhou Z., Chao X., Ju K., Wang S., Li Y. (2024). Therapeutic target genes and regulatory networks of gallic acid in cervical cancer. Front. Genet..

[25] Jin Z., Peng F., Zhang C., Tao S., Xu D., Zhu Z. (2023). Expression, regulating mechanism and therapeutic target of KIF20A in multiple cancer. Heliyon.

[26] Chen S., Zhao L., Liu J., Han P., Jiang W., Liu Y., Hou J., Wang F., Li J. (2024). Inhibition of KIF20A enhances the immunotherapeutic effect of hepatocellular carcinoma by enhancing c-Myc ubiquitination. Cancer Lett..

[27] Zhang H., Lan X., Cai L., Gao X., Gao F., Yu D., Zhang J., Zhang J., Tai Q. (2025). Tumor-associated bacteria activate PRDX1-driven glycolysis to promote immune evasion and PD-1 antibody resistance in hepatocellular carcinoma. Front. Microbiol..

[28] Kumagai S., Koyama S., Itahashi K., Tanegashima T., Lin Y.T., Togashi Y., Kamada T., Irie T., Okumura G., Kono H. (2022). Lactic acid promotes PD-1 expression in regulatory T cells in highly glycolytic tumor microenvironments. Cancer Cell.

[29] Watson M.J., Vignali P.D.A., Mullett S.J., Overacre-Delgoffe A.E., Peralta R.M., Grebinoski S., Menk A.V., Rittenhouse N.L., DePeaux K., Whetstone R.D. (2021). Metabolic support of tumour-infiltrating regulatory T cells by lactic acid. Nature.

[30] Quinn W.J., Jiao J., Teslaa T., Stadanlick J., Wang Z., Wang L., Akimova T., Angelin A., Schäfer P.M., Cully M.D. (2020). Lactate Limits T Cell Proliferation via the NAD(H) Redox State. Cell Rep..

[31] Chen P., Zuo H., Xiong H., Kolar M.J., Chu Q., Saghatelian A., Siegwart D.J., Wan Y. (2017). Gpr132 sensing of lactate mediates tumor-macrophage interplay to promote breast cancer metastasis. Proc. Natl. Acad. Sci. USA.

[32] Ji Y., Xu Z., Tang L., Huang T., Mu X., Ni C., Tang B., Lu H., Zhang C., Yang S., Wang X. (2025). O-GlcNAcylation of YBX1 drives a glycolysis-histone lactylation feedback loop in hepatocellular carcinoma. Cancer Lett..

[33] Rabinowitz J.D., Enerbäck S. (2020). Lactate: the ugly duckling of energy metabolism. Nat. Metab..

[34] Brown T.P., Ganapathy V. (2020). Lactate/GPR81 signaling and proton motive force in cancer: Role in angiogenesis, immune escape, nutrition, and Warburg phenomenon. Pharmacol. Ther..

[35] Daw C.C., Ramachandran K., Enslow B.T., Maity S., Bursic B., Novello M.J., Rubannelsonkumar C.S., Mashal A.H., Ravichandran J., Bakewell T.M. (2020). Lactate Elicits ER-Mitochondrial Mg2+ Dynamics to Integrate Cellular Metabolism. Cell.

[36] Lundø K., Trauelsen M., Pedersen S.F., Schwartz T.W. (2020). Why Warburg Works: Lactate Controls Immune Evasion through GPR81. Cell Metab..

[37] Certo M., Tsai C.-H., Pucino V., Ho P.C., Mauro C. (2021). Lactate modulation of immune responses in inflammatory versus tumour microenvironments. Nat. Rev. Immunol..

[38] Chen S., Xu Y., Zhuo W., Zhang L. (2024). The emerging role of lactate in tumor microenvironment and its clinical relevance. Cancer Lett..

[39] Gao X., Pang C., Fan Z., Wang Y., Duan Y., Zhan H. (2024). Regulation of newly identified lysine lactylation in cancer. Cancer Lett..

[40] Li L., Dong J., Xu C., Wang S. (2025). Lactate drives senescence-resistant lineages in hepatocellular carcinoma via histone H2B lactylation of NDRG1. Cancer Lett..

[41] Huang H., Wang S., Xia H., Zhao X., Chen K., Jin G., Zhou S., Lu Z., Chen T., Yu H. (2024). Lactate enhances NMNAT1 lactylation to sustain nuclear NAD(+) salvage pathway and promote survival of pancreatic adenocarcinoma cells under glucose-deprived conditions. Cancer Lett..

[42] Jiang C., He X., Chen X., Huang J., Liu Y., Zhang J., Chen H., Sui X., Lv X., Zhao X. (2025). Lactate accumulation drives hepatocellular carcinoma metastasis through facilitating tumor-derived exosome biogenesis by Rab7A lactylation. Cancer Lett..

[43] Dall'olio F.G., Marabelle A., Caramella C., Garcia C., Aldea M., Chaput N., Robert C., Besse B. (2022). Tumour burden and efficacy of immune-checkpoint inhibitors. Nat. Rev. Clin. Oncol..

[44] Du S.-S., Chen G.-W., Yang P., Chen Y.X., Hu Y., Zhao Q.Q., Zhang Y., Liu R., Zheng D.X., Zhou J. (2022). Radiation Therapy Promotes Hepatocellular Carcinoma Immune Cloaking via PD-L1 Upregulation Induced by cGAS-STING Activation. Int. J. Radiat. Oncol. Biol. Phys..

[45] Zheng G., Shi J., LI Q., Jin X., Fang Y., Zhang Z., Cao Q., Zhu L., Shen J. (2024). BAP1 inactivation promotes lactate production by leveraging the subcellular localization of LDHA in melanoma. Cell Death Discov..

[46] Gu J., Zhou J., Chen Q., Xu X., Gao J., Li X., Shao Q., Zhou B., Zhou H., Wei S. (2022). Tumor metabolite lactate promotes tumorigenesis by modulating MOESIN lactylation and enhancing TGF-β signaling in regulatory T cells. Cell Rep..

[47] Zappasodi R., Serganova I., Cohen I.J., Maeda M., Shindo M., Senbabaoglu Y., Watson M.J., Leftin A., Maniyar R., Verma S. (2021). CTLA-4 blockade drives loss of Treg stability in glycolysis-low tumours. Nature.

[48] Wu Z.-H., Wang Y.-X., Song J.-J., Zhao L.Q., Zhai Y.J., Liu Y.F., Guo W.J. (2024). LncRNA SNHG26 promotes gastric cancer progression and metastasis by inducing c-Myc protein translation and an energy metabolism positive feedback loop. Cell Death Dis..

[49] Ren X., Cheng Z., HE J., Yao X., Liu Y., Cai K., Li M., Hu Y., Luo Z. (2023). Inhibition of glycolysis-driven immunosuppression with a nano-assembly enhances response to immune checkpoint blockade therapy in triple negative breast cancer. Nat. Commun..

[50] Imai K., Hirata S., Irie A., Senju S., Ikuta Y., Yokomine K., Harao M., Inoue M., Tomita Y., Tsunoda T. (2011). Identification of HLA-A2-restricted CTL epitopes of a novel tumour-associated antigen, KIF20A, overexpressed in pancreatic cancer. Br. J. Cancer.

[51] Osawa R., Tsunoda T., Yoshimura S., Watanabe T., Miyazawa M., Tani M., Takeda K., Nakagawa H., Nakamura Y., Yamaue H. (2012). Identification of HLA-A24-restricted novel T Cell epitope peptides derived from P-cadherin and kinesin family member 20A. J. Biomed. Biotechnol..

[52] Tomita Y., Yuno A., Tsukamoto H., Senju S., Kuroda Y., Hirayama M., Irie A., Kawahara K., Yatsuda J., Hamada A. (2013). Identification of promiscuous KIF20A long peptides bearing both CD4+ and CD8+ T-cell epitopes: KIF20A-specific CD4+ T-cell immunity in patients with malignant tumor. Clin. Cancer Res..

[53] Asahara S., Takeda K., Yamao K., Maguchi H., Yamaue H. (2013). Phase I/II clinical trial using HLA-A24-restricted peptide vaccine derived from KIF20A for patients with advanced pancreatic cancer. J. Transl. Med..

[54] Akazawa Y., Hosono A., Yoshikawa T., Kaneda H., Nitani C., Hara J., Kinoshita Y., Kohashi K., Manabe A., Fukutani M. (2019). Efficacy of the NCCV Cocktail-1 vaccine for refractory pediatric solid tumors: A phase I clinical trial. Cancer Sci..

[55] Kida A., Mizukoshi E., Tamai T., Terashima T., Kitahara M., Arai K., Yamashita T., Fushimi K., Honda M., Kaneko S. (2018). Immune responses against tumour-associated antigen-derived cytotoxic T lymphocyte epitopes in cholangiocarcinoma patients. Liver Int..

