# Supplementary material for: Targeting KIF20A blocks lactylation modification to suppress immune escape in hepatocellular carcinoma
Source: iScience. 2026 Mar 13;29(4):115372. doi: 10.1016/j.isci.2026.115372 (PMC13053763; doi:10.1016/j.isci.2026.115372)

iScience, Volume *29*

Supplemental information

Targeting KIF20A blocks lactylation modification to suppress immune escape in hepatocellular carcinoma

Shujia Chen, Lili Zhao, Tongguo Miao, Ping Han, Jie Liu, Jiancun Hou, Qiang Zhao, Fengmei Wang, and Jia Li

Supplementary Table 1. The relationship between KIF20A expression in HCC and the clinicopathological characteristics of enrolled patients

Characteristics		Patients (N = 89)
Age	≤50	25 (28)
	> 50	64 (72)
Gender	Male	38 (42.7)
	Female	51 (57.3)
ALT (U/L)	≤50	56 (62.9)
	> 50	33 (37.1)
AST (U/L)	≤50	54 (60.7)
	> 50	35 (39.3)
GGT(U/L)	≤45	51 (57.3)
	> 45	38 (42.7)
AFP (ng/ml)	≤400	52 (58.4)
	> 400	37 (41.6)
HBsAg	Negative	27 (30.3)
	Positive	62 (69.7)
HBeAg	Negative	19 (21.3)
	Positive	70 (78.7)
Tumor differentiation	I-II	54 (60.7)
	III-IV	35 (39.3)
Tumor size (cm)	≤5	42 (47.2)
	> 5	47 (52.8)
BCLC stage	A	49 (47.2)
	B-C	40 (52.8)
TNM stage	I-II	56 (62.9)
	III-IV	33 (37.1)

Abbreviations: HBAg, hepatitis B surface antigen; ALT, alanine aminotransferase; AFP, alpha-Fetoprotein; BCLC, Barcelona Clinic Liver Cancer staging.

Supplementary Table 2. Nucleotide target / Primers sequences.

Amplicons	Sequences (5'-3')
si-KIF20A-Ctrl (F)	TGGCACGTCTTCGGACCTA
si-KIF20A-Ctrl (R)	TTCTTGCGTACCACAGACCC
si-KIF20A (F)	GCTTGCTGTCCGATGACGAT
si-KIF20A (R)	ACGGACACAACCCTGATCTT
si-c-Myc-Ctrl (F)	GGAAAACCAGCCTCCCGC
si-c-Myc-Ctrl (R)	CTGCTGCTGCTGGTAGAAGT
si-c-Myc (F)	CCGCTTCTCTGAAAGGCTCT
si-c-Myc (R)	CCGCTTCTCTGAAAGGCTCT
si-LDHA-Ctrl (F)	CCGCCGATTCCGGATCTCAT
si-LDHA-Ctrl (R)	AGGTCAAGATATCCACTTTGCCA
si-LDHA (F)	CGCCGATTCCGGATCTCA
si-LDHA (R)	GGTCAAGATATCCACTTTGCCA
si-LDHB -Ctrl (F)	CCTTTAGCTCTGAGCATCCG
si-LDHB-Ctrl (R)	GTTCATCAGCCAGAGACTTTCC
si-LDHB (F)	CGCGGCCTTTAGCTCTGA
si-LDHB(R)	TTCATCAGCCAGAGACTTTCC
sh-LDHA	CCGG-CGTACACCATTCAAGGTACTA-<br>CTCGAGTAGTACCTTGAATGGTGTACGTTTTTT
sh-LDHB	CCGG-GAGGAAATTTGCAACGAAATG-CTCGAG-<br>CATTTCGTTGCAAATTTCCTC-TTTTTT
c-Myc (F)	TACAACACCCGAGCAAGCAC
c-Myc (R)	CTAACGTTGAGGGGCATCGT
PD-L1 (F)	CCACTGTTAGTAGAAGAATC

PD-L1 (R)	TCCACTTCACTGTCTCAG
β-actin (F)	AATGGGCAGCCGTTAGGAAA
β-actin (R)	GCCCAATACGACCAAATCAGAG
KIF20A promoter-1 (F)	CACCATGTTAGCCAGGATG
KIF20A promoter-1 (R)	ACGGTCTGAGAAGGTAGAT
KIF20A promoter-2 (F)	AGGCCCAGTATTCCTGTCTGG
KIF20A promoter-2 (R)	GCACTCAGTACTGTCTCCT
KIF20A promoter-Luc (F)	TTGGTATTTTTATTAGAGACGG
KIF20A promoter-Luc (R)	CCTGGAGTTGCGAGGGGACA
PD-L1 promoter (F)	CAAGCTGTCCAATCAATAGCTG
PD-L1 promoter (R)	CCAGAAGAGCCAAGGAAACGTC
KIF20A (F)	TGGCACGTCTTCGGACCTA
KIF20A (R)	TTCTTGCGTACCACAGACCC
c-Myc (F)	TACAACACCCGAGCAAGCAC
c-Myc (R)	CTAACGTTGAGGGGCATCGT
PD-L1(F)	CCACTGTTAGTAGAAGAATC
PD-L1 (R)	TCCACTTCACTGTCTCAG
β-actin(F)	AATGGGCAGCCGTTAGGAAA
β-actin(R)	GCCCAATACGACCAAATCAGAG
KIF20A promoter-1 (F)	CACCATGTTAGCCAGGATG
KIF20A promoter-1 (R)	ACGGTCTGAGAAGGTAGAT
KIF20A promoter-2 (F)	AGGCCCAGTATTCCTGTCTGG
KIF20A promoter-2 (R)	GCACTCAGTACTGTCTCCT
KIF20A promoter-Luc (F)	TTGGTATTTTTATTAGAGACGG
KIF20A promoter-Luc (R)	CCTGGAGTTGCGAGGGGACA

PD-L1 promoter (F)	CAAGCTGTCCAATCAATAGCTG
PD-L1 promoter (R)	CCAGAAGAGCCAAGGAAACGTC
Human KIF20A-KO-sg	GCTGATGAGCTACGAGTACCG

Data S1: Uncropped Western Blot images

Figure 1

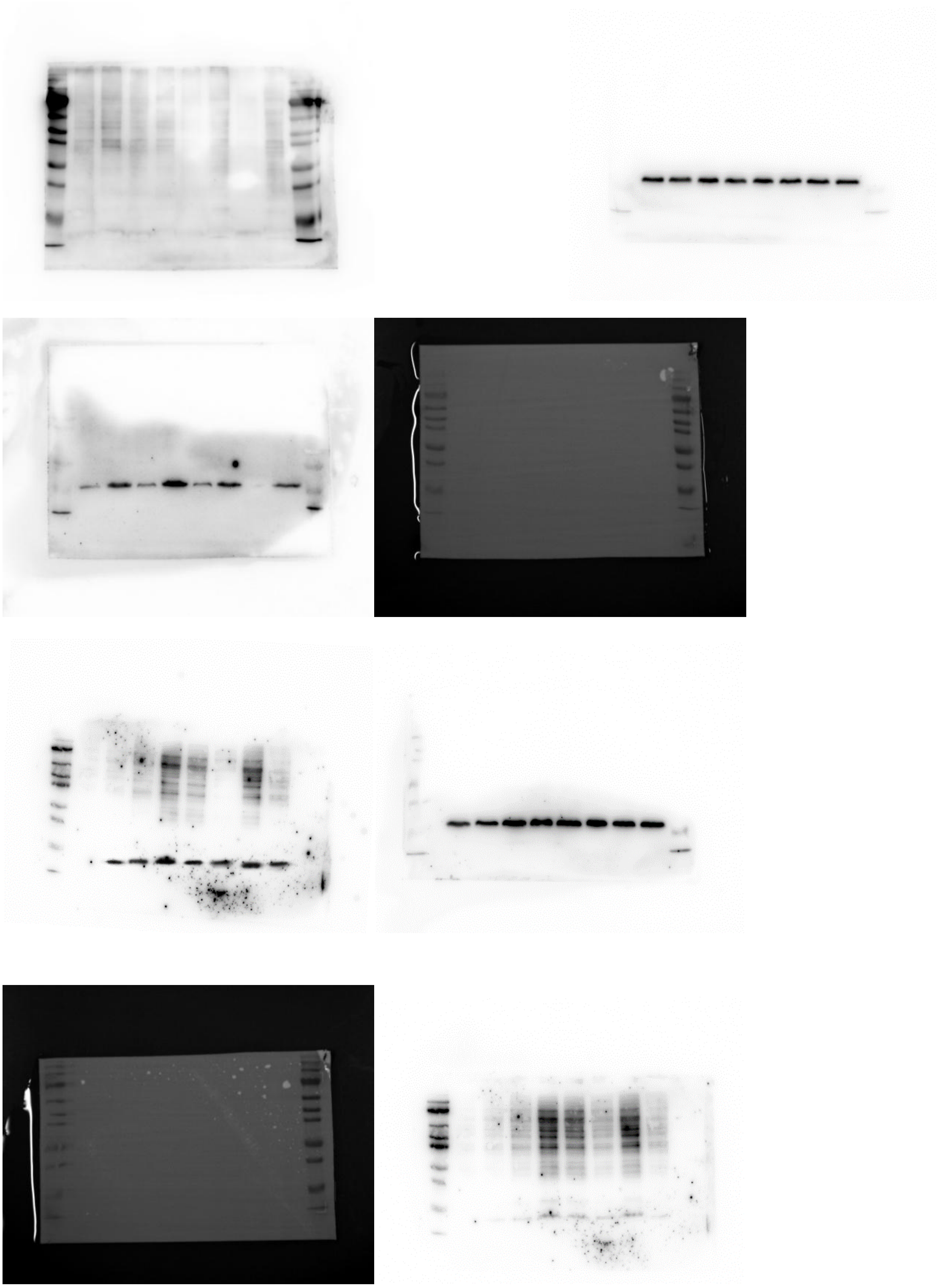

Figure 2

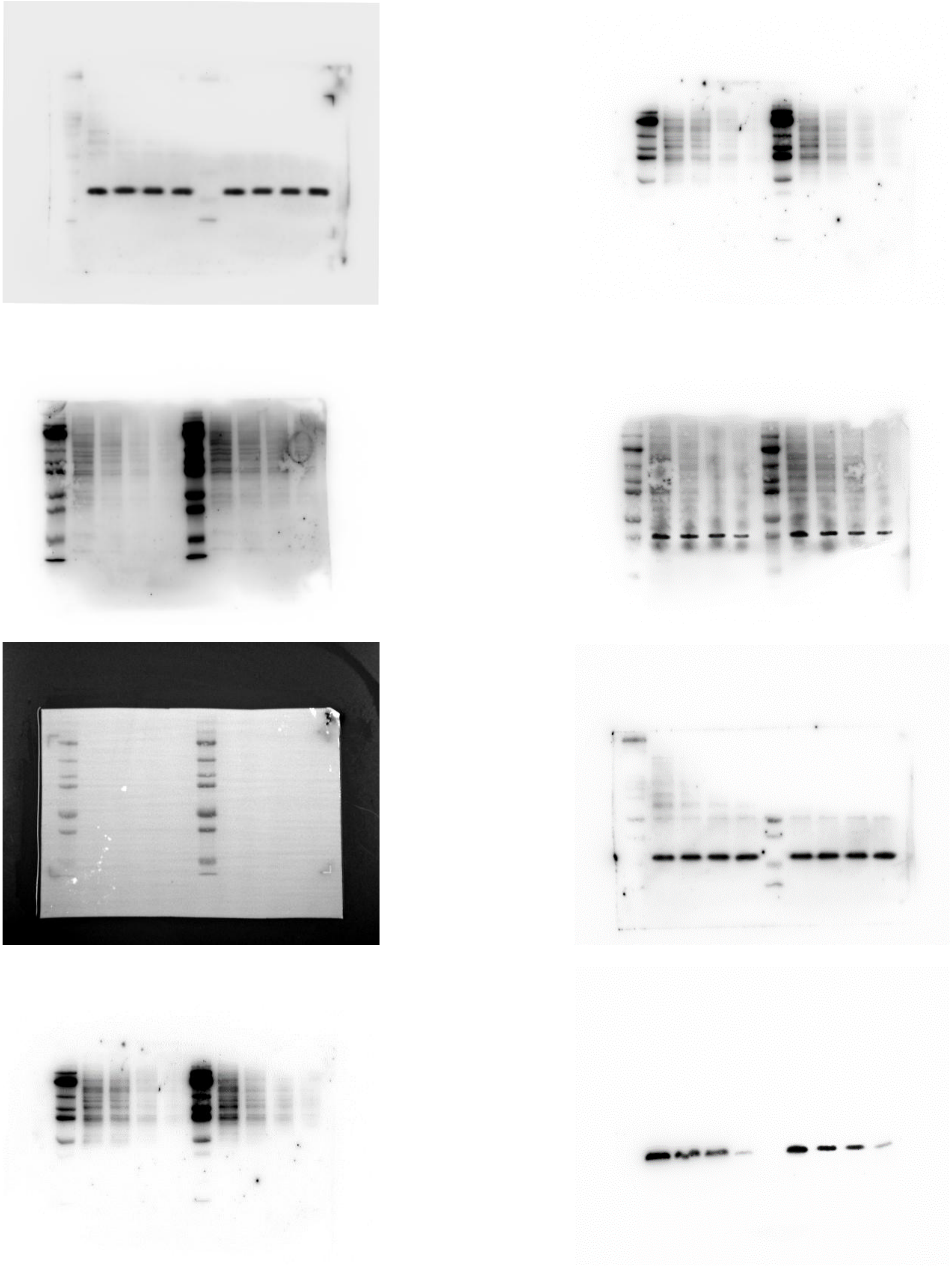

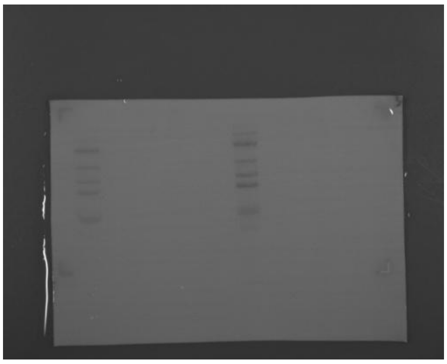

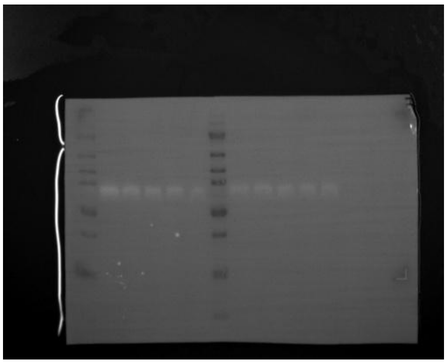
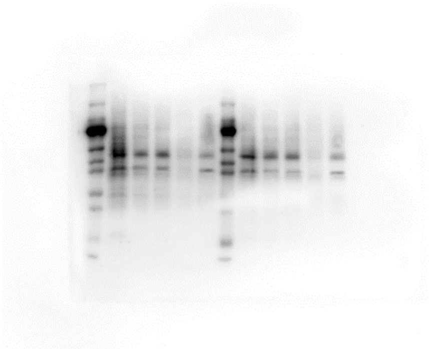
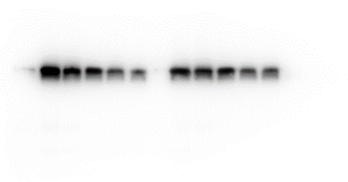
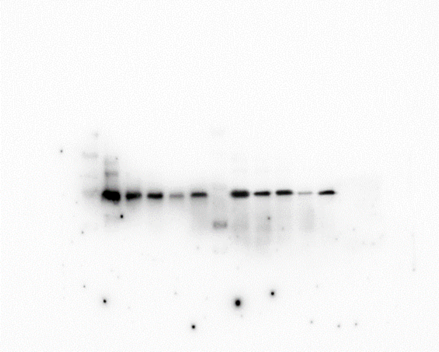
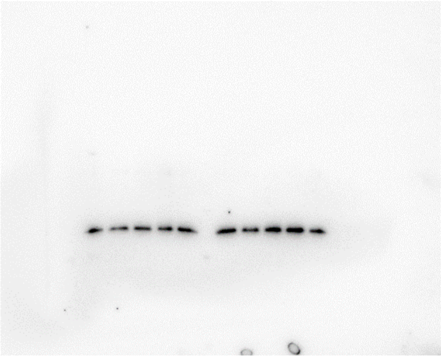

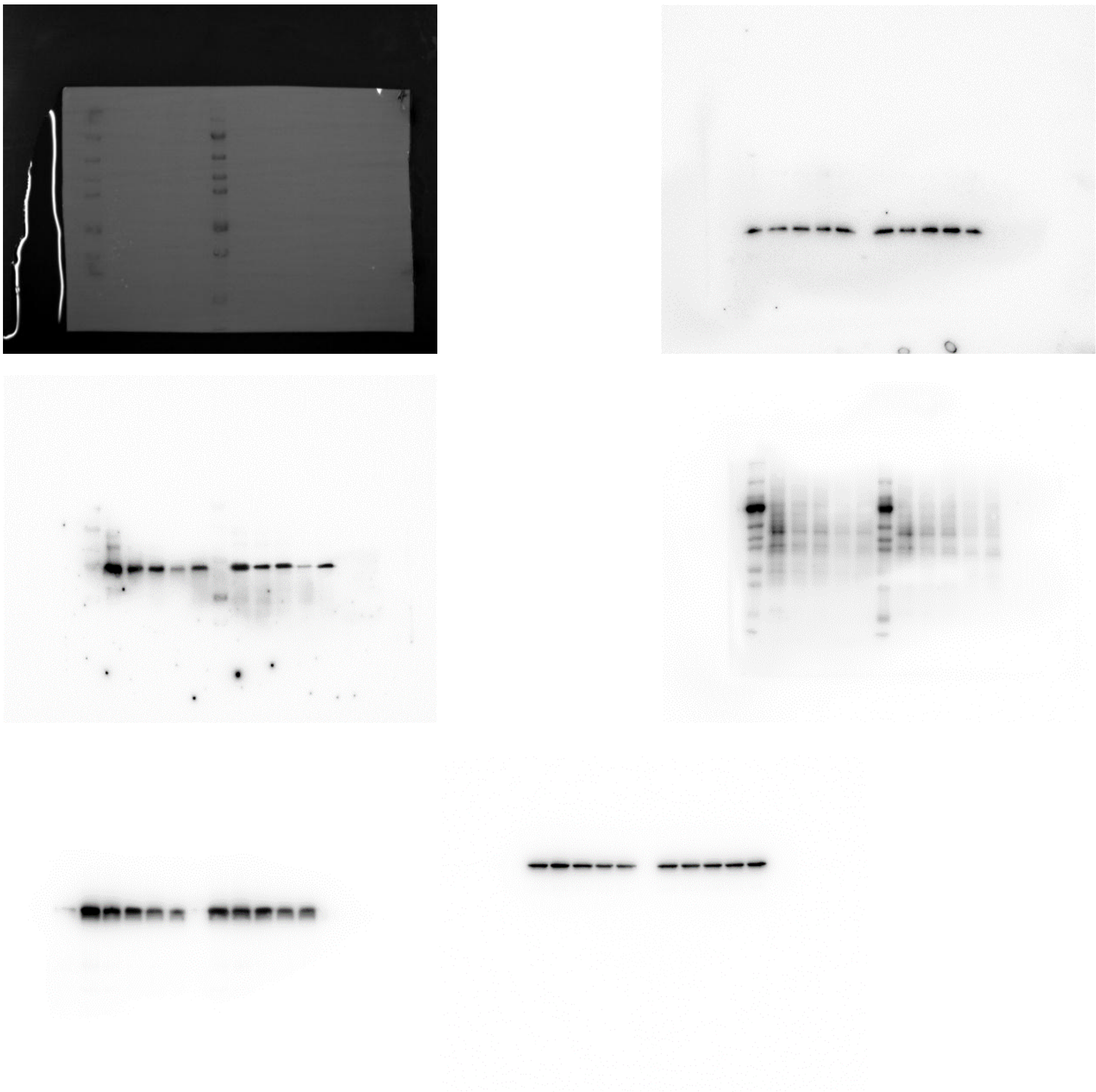

Figure 3

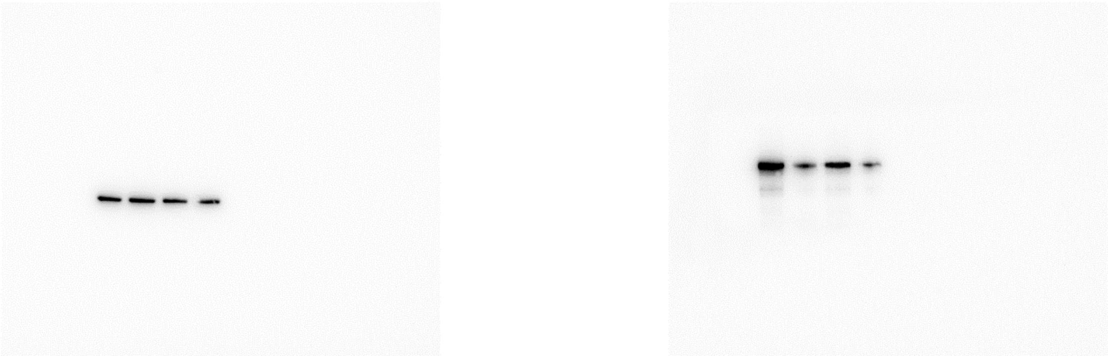

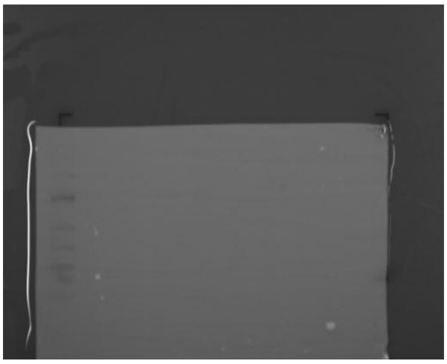

Figure 4

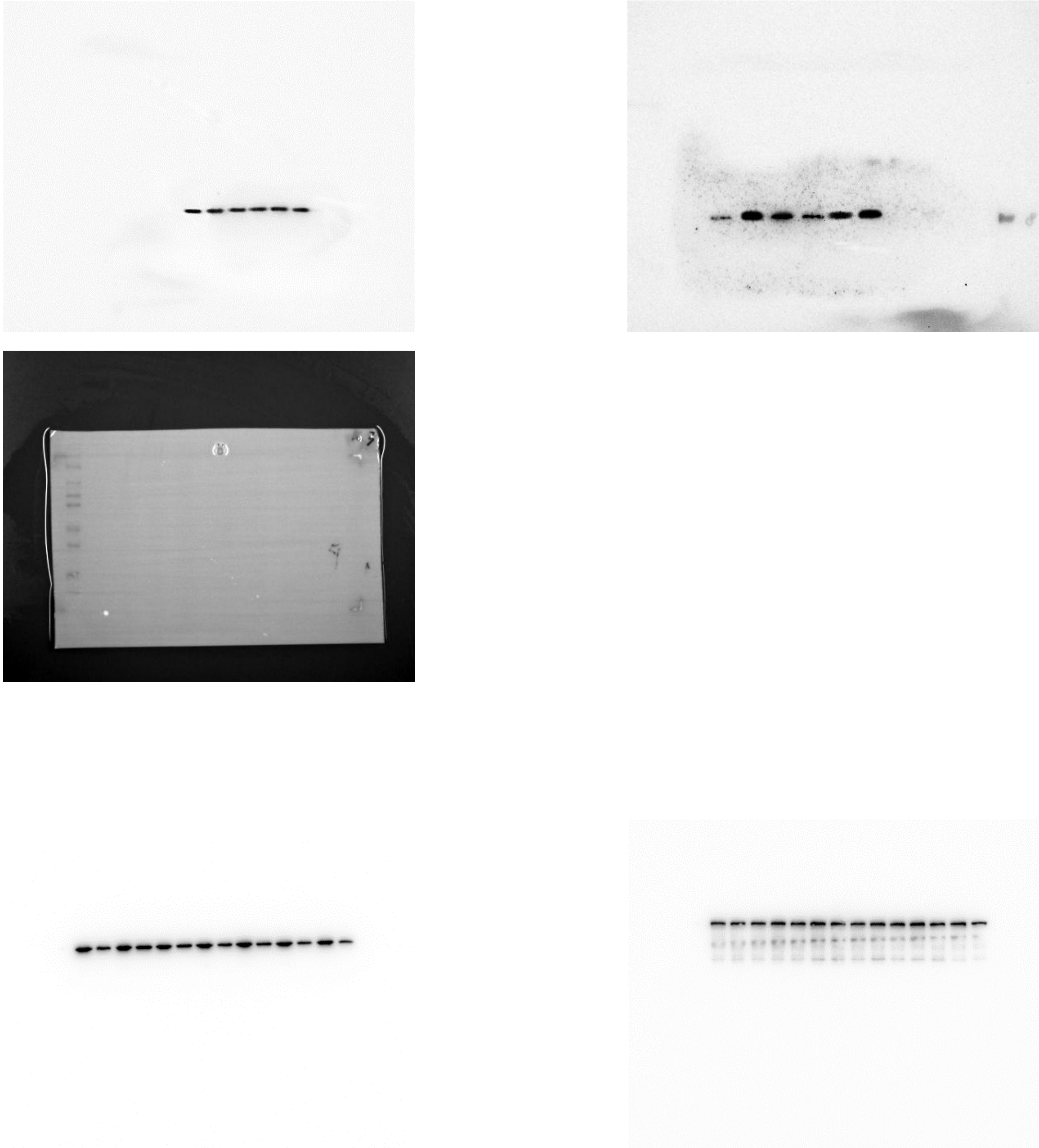

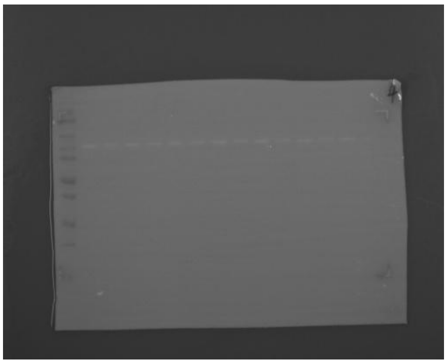

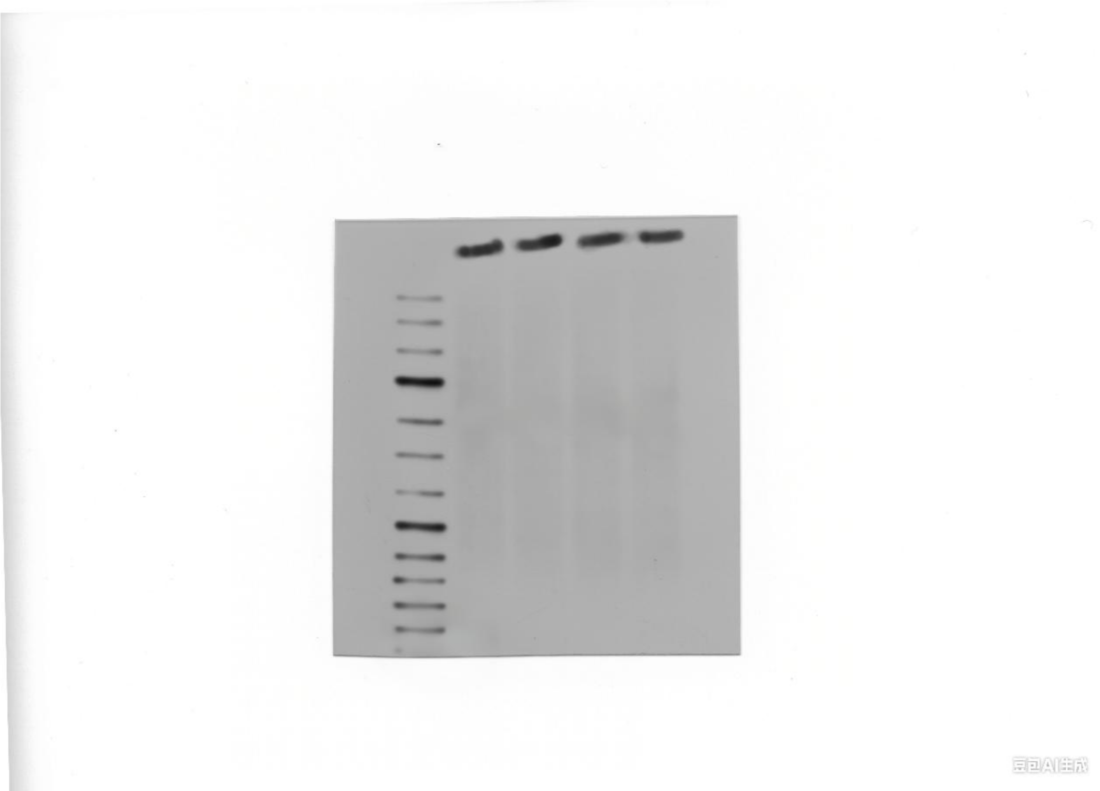
豆包AI生成

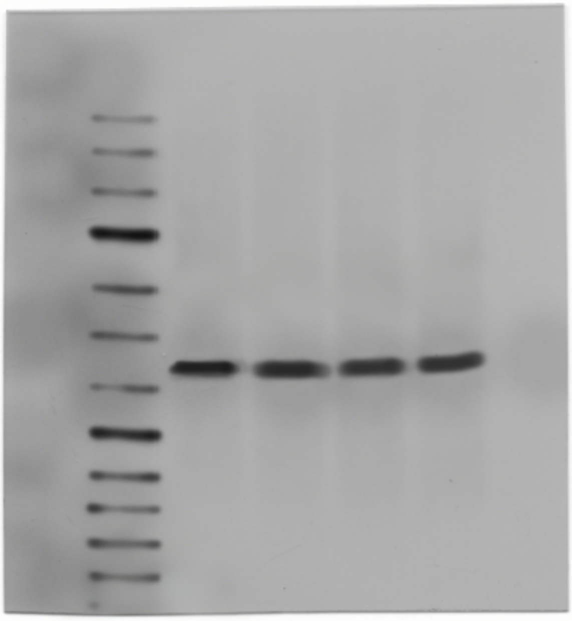

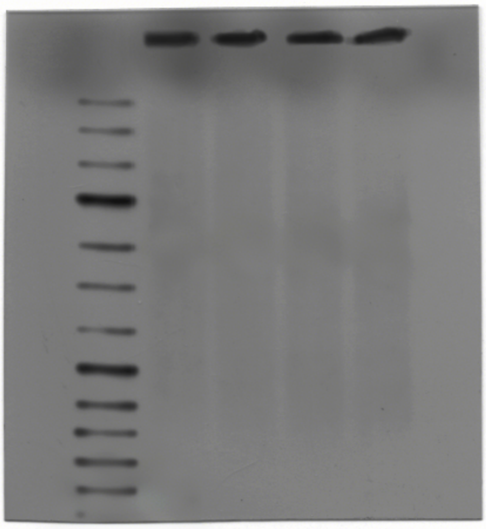

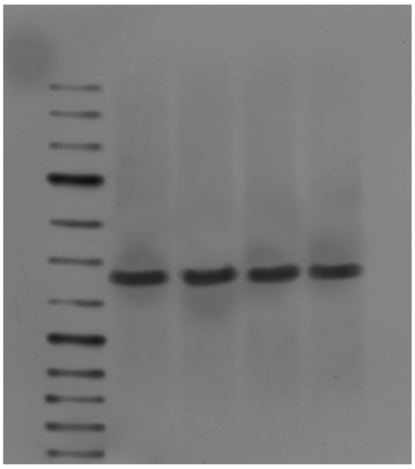

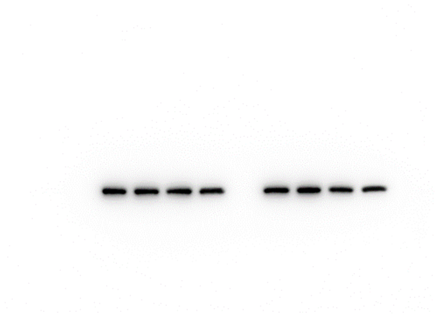

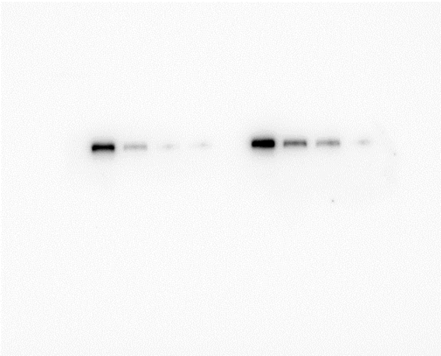

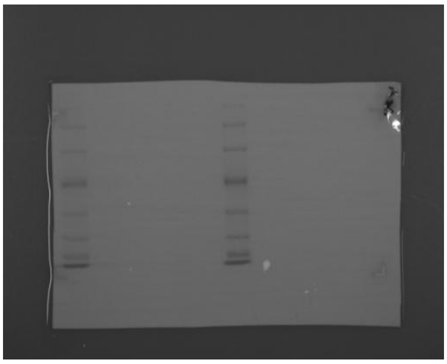

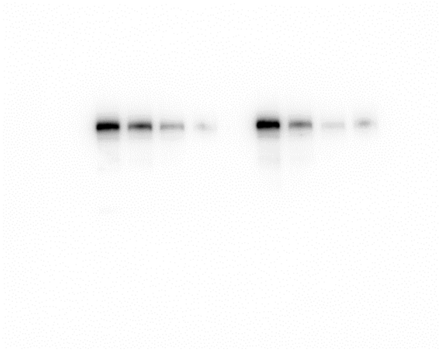

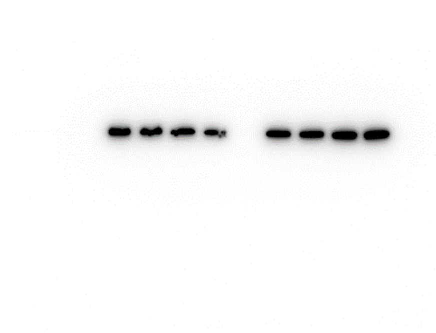

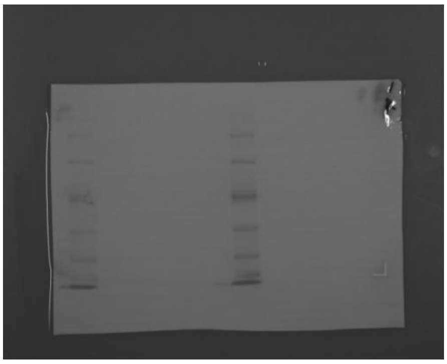

Figure 5

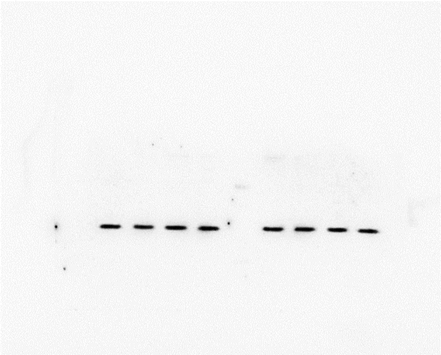

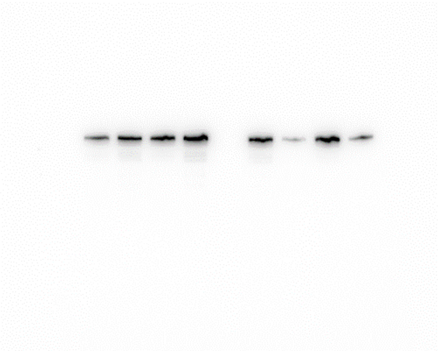

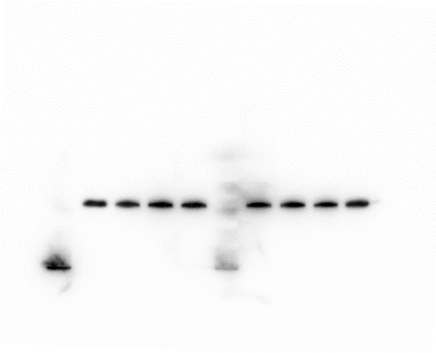
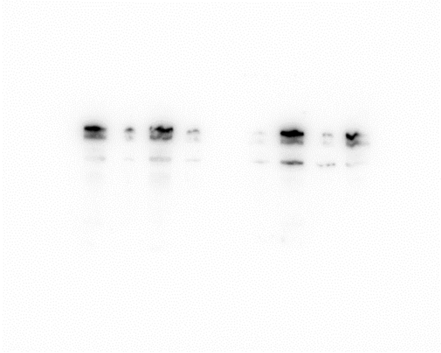
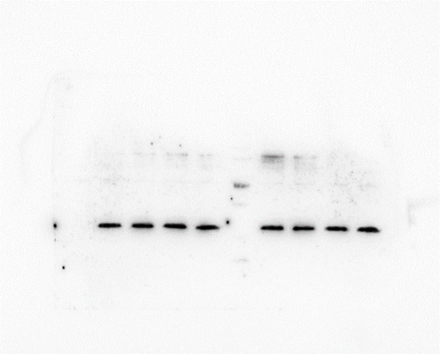
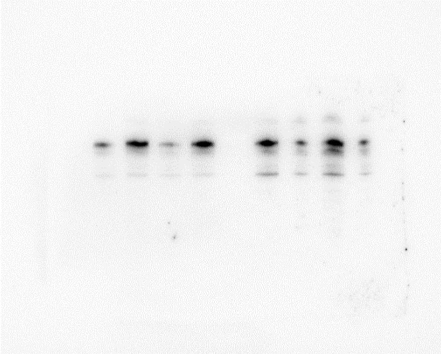
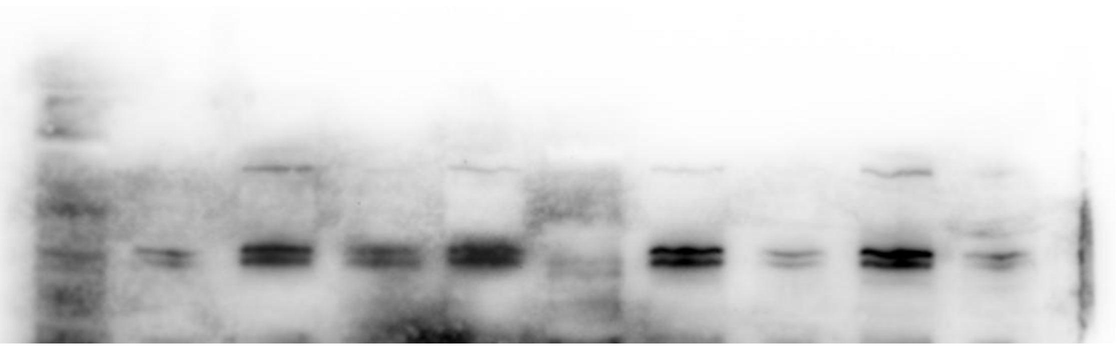
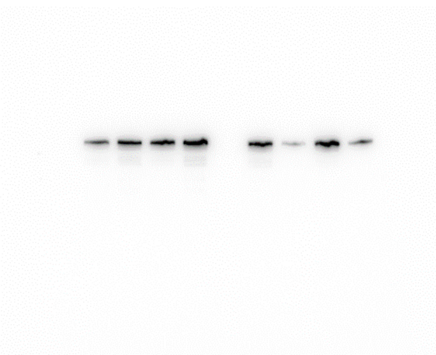
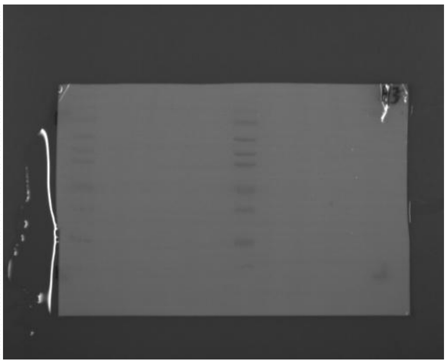

Figure 6

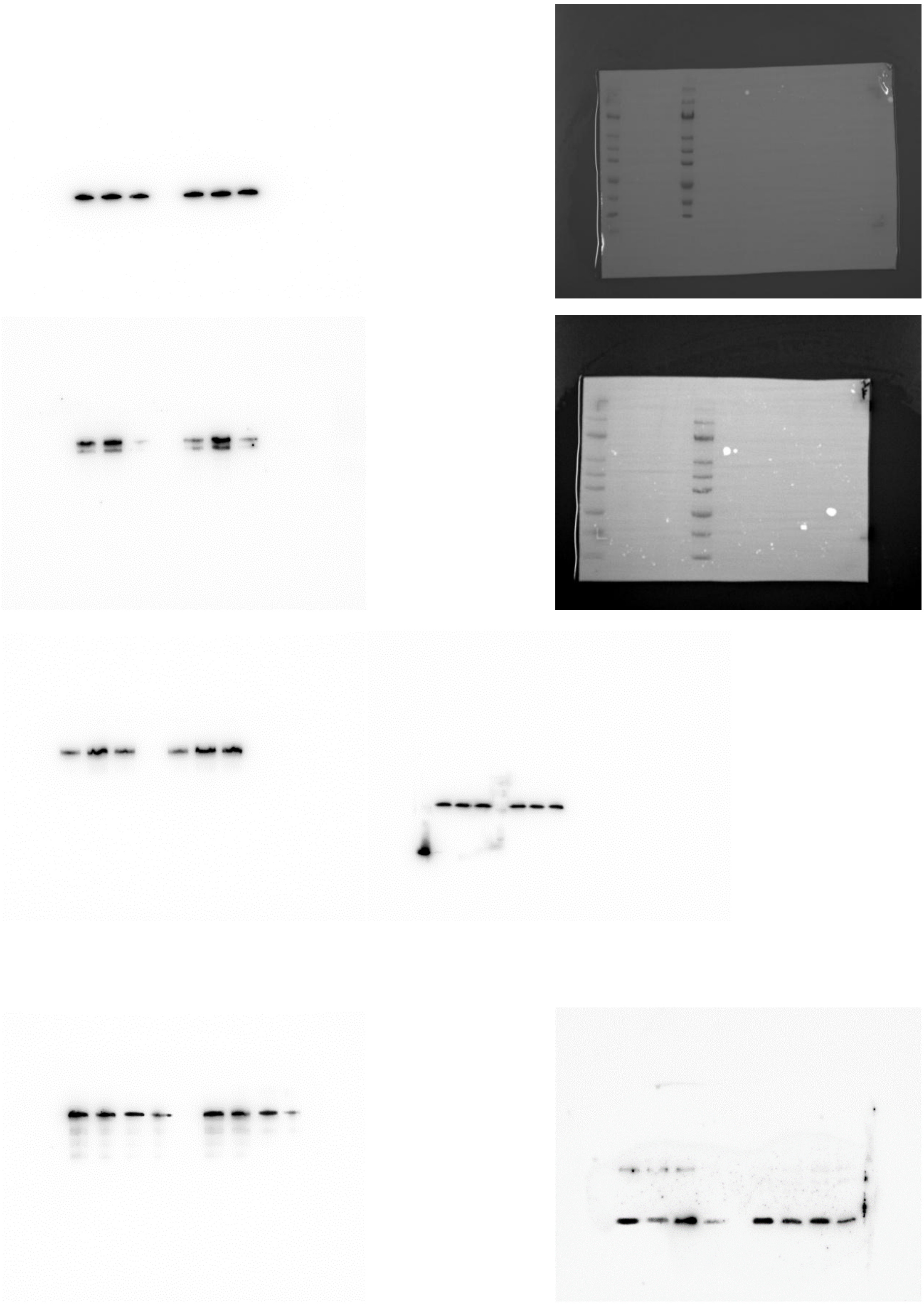

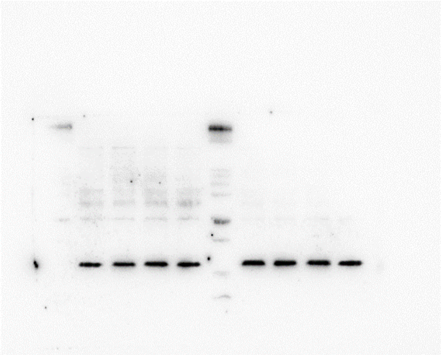

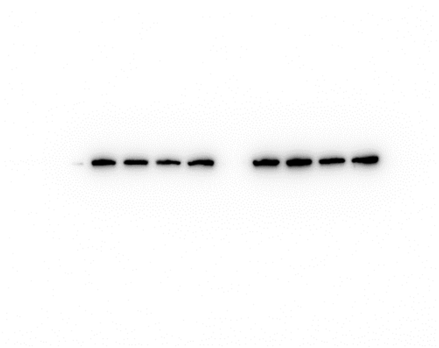

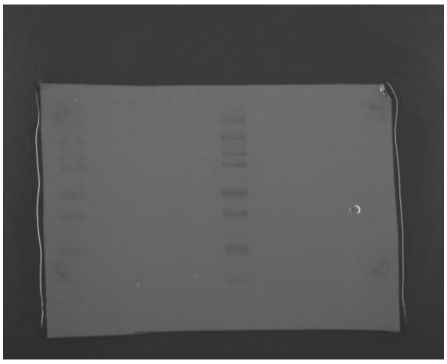

Figure 7

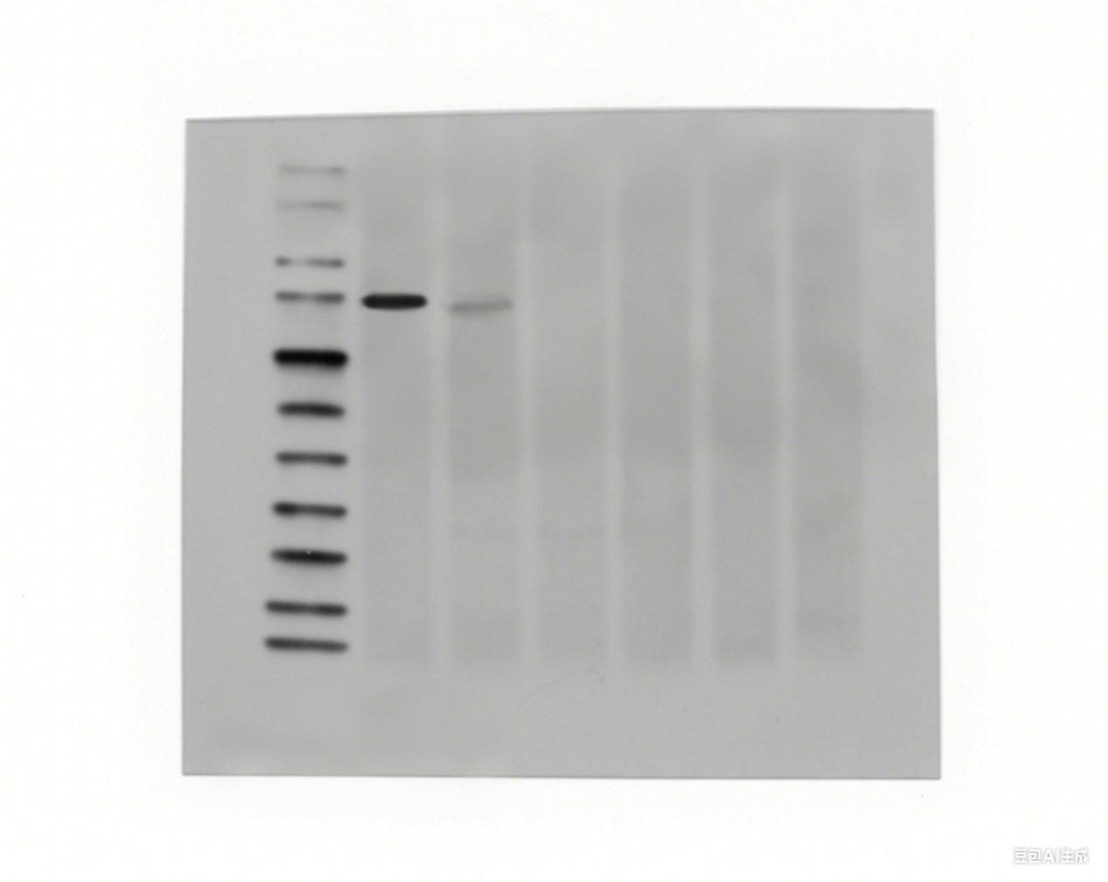

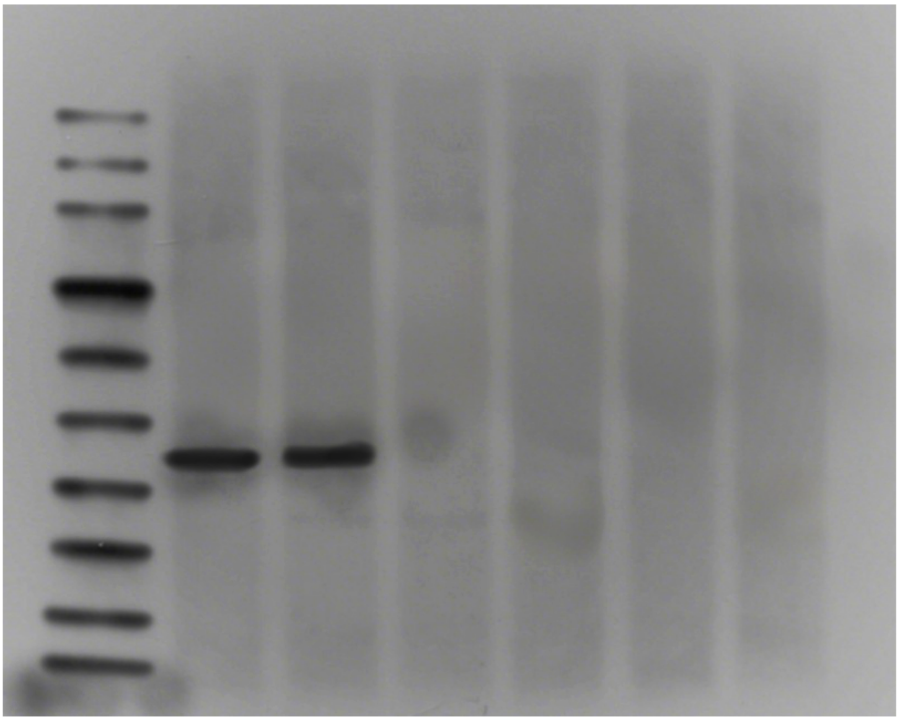

Supplement: Document S1. Tables S1 and S2 and Data S1 [file mmc1.pdf]
